# Mapping Ferroelectric Fields Reveals the Origins of the Coercivity Distribution

**DOI:** 10.1021/acsnano.4c04526

**Published:** 2024-07-17

**Authors:** Ho Leung Chan, Shelby S. Fields, Yueyun Chen, Tristan P. O’Neill, Megan K. Lenox, William A. Hubbard, Jon F. Ihlefeld, Brian C. Regan

**Affiliations:** † Department of Physics and Astronomy, University of California, Los Angeles, California 90095, United States; ‡ California NanoSystems Institute, University of California, Los Angeles, California 90095, United States; § Department of Materials Science and Engineering, University of Virginia, Charlottesville, Virginia 22904, United States; ∥ NanoElectronic Imaging, Inc., Los Angeles, California 90095, United States; ⊥ Charles L. Brown Department of Electrical and Computer Engineering, University of Virginia, Charlottesville, Virginia 22904, United States

**Keywords:** ferroelectric, hafnium zirconium oxide, transmission electron microscopy, electron beam-induced current, nonvolatile memory, depolarization field, imprint

## Abstract

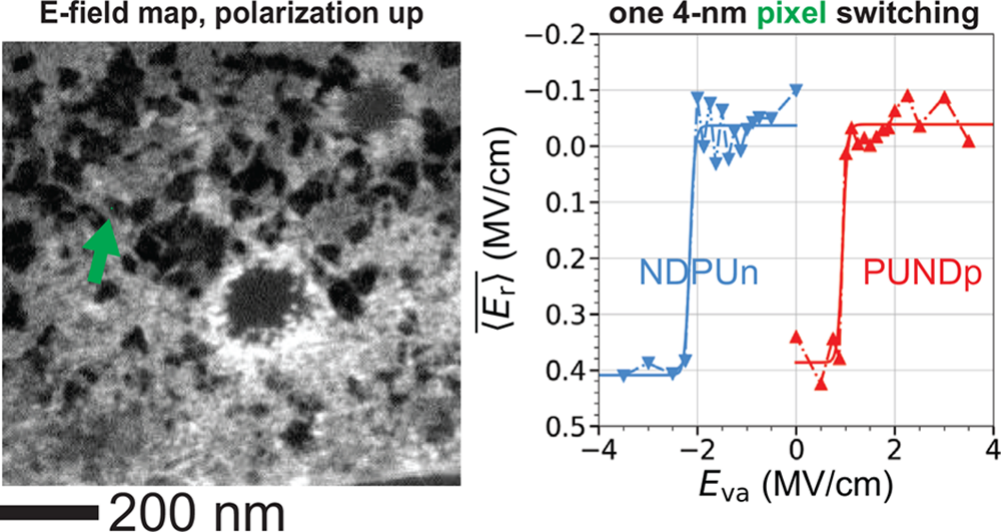

Better techniques for imaging ferroelectric polarization would aid the development of new ferroelectrics and the refinement of old ones. Here we show how scanning transmission electron microscope (STEM) electron beam-induced current (EBIC) imaging reveals ferroelectric polarization with obvious, simply interpretable contrast. Planar imaging of an entire ferroelectric hafnium zirconium oxide (Hf_0.5_Zr_0.5_O_2_, HZO) capacitor shows an EBIC response that is linearly related to the polarization determined *in situ* with the positive-up, negative-down (PUND) method. The contrast is easily calibrated in MV/cm. The underlying mechanism is magnification-independent, operating equally well on micrometer-sized devices and individual nanoscale domains. Coercive-field mapping reveals that individual domains are biased “positive” and “negative”, as opposed to being “easy” and “hard” to switch. The remanent background *E*-fields generating this bias can be isolated and mapped. Coupled with STEM’s native capabilities for structural identification, STEM EBIC imaging provides a revolutionary tool for characterizing ferroelectric materials and devices.

Ferroelectrics have been proposed for use in a wide range of transformative applications, ranging from next-generation computer memory^1−7^ to actuators, sensors, high-frequency filters, and environmental energy harvesters.^8,9^ However, the gap between the material properties that might be possible in principle and those that are realized in practice has limited actual implementations.^1−9^ To optimize ferroelectric materials and thereby narrow this gap, imaging techniques capable of measuring ferroelectric response are invaluable.^10−29^

Because of the fundamental connections between atomic-scale structure and ferroelectric function, high-resolution imaging is particularly useful. Piezoresponse force microscopy (PFM)^10−18^ and transmission electron microscopy (TEM)^19−27^ are the standard options. PFM maps ferroelectric domains via their electromechanical response. However, this response is difficult to calibrate^12,18^ and is typically reported in arbitrary units, as opposed to units relevant to ferroelectricity (e.g., MV/cm or μC/cm^2^). PFM is often conducted on the bare surface of the ferroelectric, which results in differences in the electrical, mechanical, and environmental boundary conditions from those in real devices.^14^ Imaging through an electrode can be performed, but with degraded spatial resolution. Distinguishing actual ferroelectricity from hysteretic tip–sample electrostatic interactions and charge injection is also challenging.^15,16^ And the ≳5–10 nm resolution of PFM limits its ability to identify the unit-cell-scale defects and underlying crystallographic structures that govern the macroscopic ferroelectric response.^10,19^

In contrast to PFM, high-resolution TEM excels at characterizing crystal structure and defects, but it is not particularly adept at measuring ferroelectric polarization. In single crystals, diffraction-contrast (S)TEM can distinguish alternately polarized domains and thus track domain wall motion,^19−22^ but it cannot quantitatively determine polarization or the remanent electric field. High-resolution (scanning) (S)TEM can infer local polarization fields from precision measurements of atomic positions, but the shifts are difficult to see in the raw data and the resulting polarization can only be quantified with the help of detailed simulations.^23−27^ Disentangling the atomic electric fields and the mesoscopic polarization field is challenging and, due to dynamical scattering effects, extremely sensitive to experimental parameters such as sample thickness and tilt.^24^ Additionally, the atomic-resolution requirement necessarily restricts this technique to small fields-of-view on zone axis, which is problematic with polycrystalline samples.

STEM electron beam-induced current (EBIC) imaging suffers from none of these limitations. It has access to all of the usual structural determination powers of standard STEM, but it does not require atomic-resolution imaging. It is not sensitive to sample thickness or tilt. The operative contrast mechanism is independent of magnification, so it can equally well characterize whole devices or individual domains. Similarly, there is no requirement that imaging be done on zone axis, so single-crystal and polycrystalline samples are equally easy to investigate. STEM EBIC imaging naturally distinguishes between atomic and mesoscopic fields by answering the question that is ultimately relevant: which way do test charges inserted in the sample go? Polarization is readily apparent in the raw data, and remanent fields can be quantified with the aid of straightforward calibration measurements. STEM EBIC imaging determines both the electric field *E* and, when coupled with traditional transport, the displacement field *D*. It thereby determines the polarization *P* (via **D** = ϵ_0_**E** + **P**), providing a complete picture of the fields in the sample.

In EBIC imaging, a focused electron beam is rastered over a sample while, simultaneously, electrical currents induced in the sample are captured from one or more electrodes and digitized. Associating the measured current with the beam position produces the EBIC image.^30^ Larger EBICs are generated where *E*-fields in the sample separate electron–hole pairs generated by the beam. An EBIC image thus contains information about the magnitude and direction of internal electric fields, making EBIC imaging ideal for the study of ferroelectric materials.

EBIC imaging is most commonly implemented in a scanning electron microscope (SEM),^30^ and SEM EBIC has even been applied to the study of ferroelectric polarization.^29^ However, STEM EBIC has several important advantages over SEM EBIC.^31^ Because a STEM sample is electron-transparent, the electron beam’s interaction volume is nanometer-scale, not micrometer-scale. The smaller interaction volume improves the spatial resolution, in some cases sufficiently to resolve the atomic lattice.^32^

Here we study ferroelectric atomic layer deposited (ALD) Hf_0.5_Zr_0.5_O_2_ (HZO), which can be highly scaled and is CMOS-compatible.^2−6,33,34^ ALD HZO is polycrystalline and polymorphic, with many competing crystal phases of similar free energies.^7,34,35^ Its ferroelectric phases are presumed^36^ to be only stable in thin films, but how dopants, electrode materials, confinement, defects, strain, and size effects interact to produce ferroelectricity is still very unclear.^7,36−39^ Thus, this material is a supremely relevant and challenging target for a polarization imaging technique: it is perhaps the most promising material for next-generation nonvolatile memories,^2−6,33,34^ but understanding the relationships between its crystal structure, phase stabilizing mechanisms, and ferroelectricity has proved elusive.^7,36−39^

## Results

**Figure 1:**
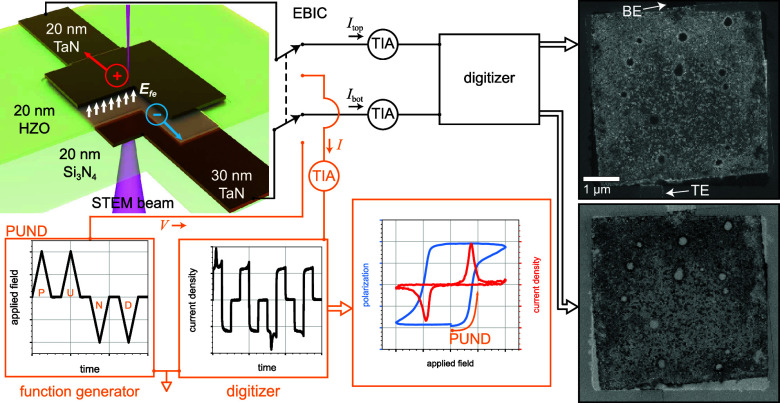
Experiment overview. While in the STEM, a microfabricated TaN/HZO/TaN capacitor on an electron-transparent Si_3_N_4_ membrane is switched between the lower and upper signal paths for PUND and EBIC measurements, respectively. The PUND sequence shown (plots) leaves the HZO polarized down (*P*_↓_). Subsequent EBIC imaging (images) maps the remanent ⟨*E*_r_⟩, which nominally points up. Electron–hole pair separation in such an electric field produces a hole current *I*_top_ (bright contrast) and an electron current *I*_bot_ (dark contrast). Transimpedance amplifiers (TIAs) convert currents to voltages for digitization. Standard STEM images not shown here (see SI) are acquired simultaneously.

We measure a 30/20/20 nm TaN/HZO/TaN capacitor’s global and local polarization responses *in situ* (Figure 1). To measure the global polarization, we use transport, specifically the positive-up, negative-down (PUND) method.^40,41^ To map the local polarization, we perform STEM EBIC imaging immediately following a PUND measurement. Since PUND leaves the HZO polarized according to the particulars of the pulse sequence just applied, PUND prepares the capacitor for the subsequent STEM EBIC measurement. We use an extended PUND sequence consisting of two maximum peak voltage PUND waveforms (“init”), two variable peak voltage PUND waveforms (“var”), and a PU waveform at the same variable voltage (“set”). “PUNDp3”, for example, indicates a PUND sequence that begins with ±7 V init pulses and concludes with two +3 V pulses, while “NDPUn3” has the opposite polarity but is otherwise identical. (See Methods for further details.)

**Figure 2:**
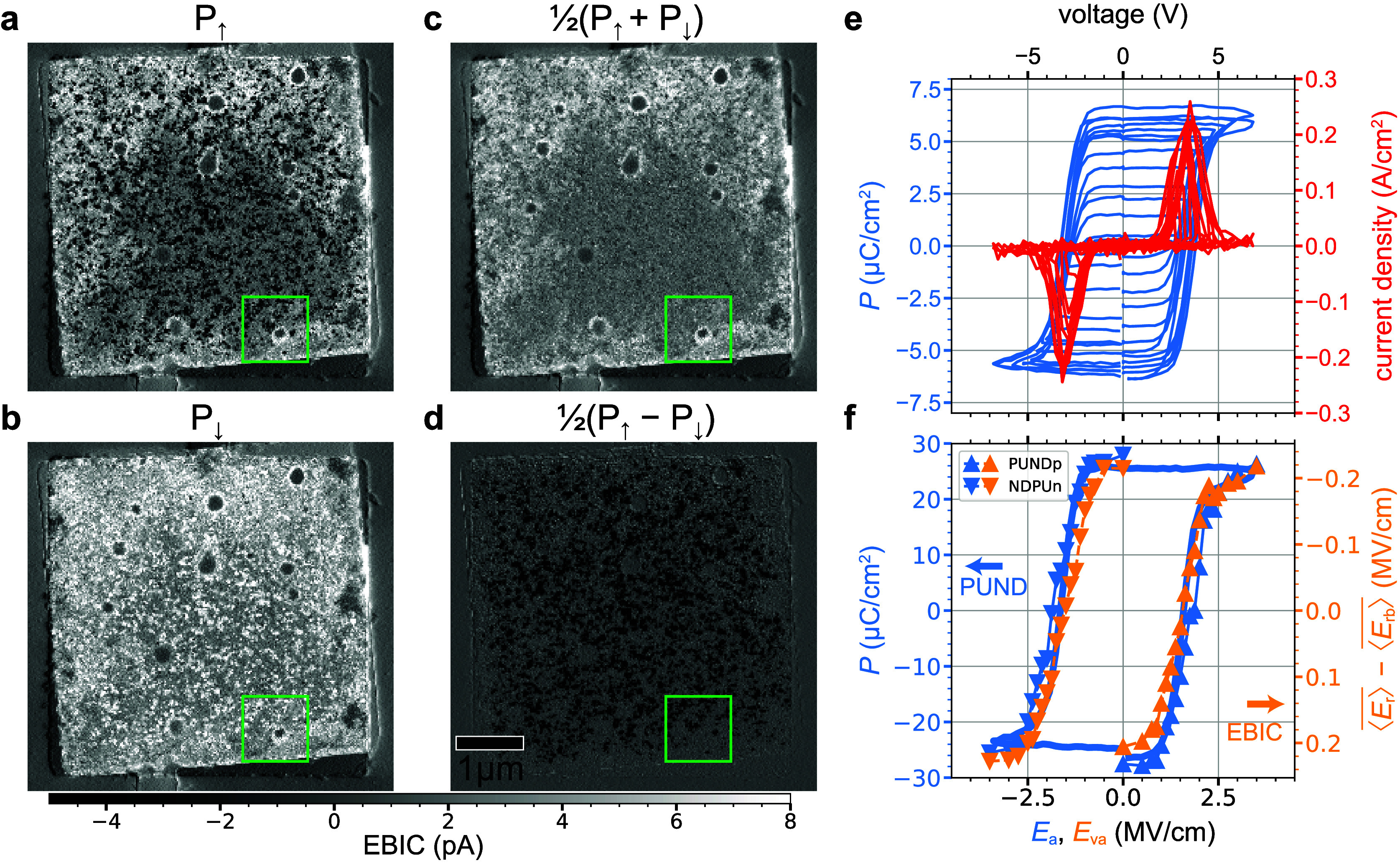
Device-scale comparison of polarization measurements: STEM EBIC vs PUND. EBIC difference (*I*_top_ – *I*_bot_)/2 images show the capacitor in the maximal *P*_↑_ (a) and *P*_↓_ (b) states. These images, which are acquired after PUNDp7 and NDPUn7 measurements, respectively (i.e., with 3.5 MV/cm peak applied fields), are the first two in a 40-point data set with var voltages varying 7–0 V (Movie M1). Polarization state sum (c) and difference (d) images highlight the pinned and the switching domains, respectively, and quantify the background remanent fields ⟨*E*_rb_⟩ and the switching remanent fields ⟨*E*_rs_⟩ inside the capacitor, respectively. The green box indicates the field of view shown in Figure 3. (e) The polarization *P* measured by PUND varies with the applied field amplitude *E*_a_. Here *P* is calculated using the PUNDp second var sequences (Figure S1, Figure S2e) and assuming that the entire capacitor (*A*_*c*_ = 22 μm^2^) switches (Figure S7c). (f) Averaging images like (c) over the switching area for each of the various var applied fields *E*_va_ gives the field , as determined by EBIC imaging. Comparing with *P*, as determined by PUND, shows them to be proportional. For (f) *P* is calculated using the actual switching area (*A*_*s*_ = 5.3 μm^2^) determined by EBIC imaging (Figure S7d).

After a PUNDp7 sequence, STEM EBIC imaging of a TaN/HZO/TaN capacitor and the surrounding region shows marked variations in the local electric fields (Figure 2a). Away from the electrodes, the contrast is neutral. The HZO in these regions is not subject to confinement strain, never polarized, and not near an EBIC collection electrode. It thus generates little EBIC (Figure S8).

The capacitor itself is in a decidedly inhomogeneous polarization state, with some regions showing dark contrast indicative of the expected *P*_↑_ state and others showing bright contrast indicative of the *P*_↓_ state. After a NDPUn7 pulse sequence has been applied, the resulting STEM EBIC image is brighter over much of the capacitor (Figure 2b), indicating that the contrast-generating remanent *E*-field ⟨*E*_r_⟩ is more positive. Standard STEM bright field (BF) and annular dark field (ADF) imaging shows no significant changes (Figure S5).

To separate the switching domains from the pinned domains, we construct sum [(*P*_↓_ + *P*_↑_)/2] and difference [(*P*_↓_ – *P*_↑_)/2] images. As Figure 2a and Figure 2b map ⟨*E*_r↑_⟩ and ⟨*E*_r↓_⟩ inside the capacitor, respectively, the sum (Figure 2c) and difference images (Figure 2d) map the remanent background fields ⟨*E*_rb_⟩ and remanent switching fields ⟨*E*_rs_⟩ inside the capacitor, respectively. By definition these fields are related by
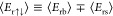
1Here“ ∓ ” appears because the remanent switching field ⟨*E*_rs_⟩ is positive by definition, and we measure it to be directed opposite the polarization state *P*_↑↓_. (Arrows denote the polarization state, never the *E*-field direction.)

The sum (or average) polarization image (Figure 2c) shows domains that are not switching at 3.5 MV/cm (see also Figure S11). These domains, clearly visible in the EBIC images, cannot be detected with PUND. Such global transport methods can only infer that pinned domains might exist based on a less-than-ideal polarization response, for they have no way to distinguish pinned domains from unpolarized or nonferroelectric material. PUND is also a “destructive” technique, in that it must switch the polarization in order to measure it. STEM EBIC imaging maps built-in *E*-fields nondestructively.

The pinned domains are mostly in the *P*_↓_ (bright) state. While we cannot explain why this state is preferred, the existence of an asymmetry is unsurprising.^42^ The interfaces are inherently asymmetric, because depositing HZO on TaN is not the same as depositing TaN on HZO. This interface asymmetry is likely the root cause of the pinning asymmetry.

A smattering of small (≲ 100 nm) roundish regions that show little EBIC also dot the capacitor (Figure S5). No sign of these spots appears in the standard bright field (BF) and annular dark field (ADF) STEM images collected simultaneously (Figures S5 and S6). These defects appear to be regions where the TE does not make good contact to the HZO, perhaps because of gas bubbles or some contamination that was on the HZO when the TE was deposited during device fabrication. STEM EBIC imaging is here revealing connectivity defects,^31^ which can be as important as materials defects for understanding device function.

The polarization difference image (Figure 2d) shows the domains that are switching at 3.5 MV/cm with dark contrast. These domains are, of course, entirely responsible for the currents measured by PUND (Figure 2e). To determine how well the polarization imaged with STEM EBIC compares to that measured with PUND, we perform sequential PUNDp and NDPUn measurements with a series of var voltages ranging from 7 to 0 V (Movie M1). After every transport measurement (Figure 2e) we acquire an EBIC image of the whole capacitor. As the var voltage decreases, the PUNDp (NDPUn) series images show less and less of the *P*_↑_ (*P*_↓_) state characteristic of the var voltage polarity, and more and more of the *P*_↓_ (*P*_↑_) state characteristic of the init voltage polarity.

We seek to quantify the switching remanent field ⟨*E*_rs_⟩ characteristic of ideal HZO. We therefore average the EBIC over just the areas that are switchable (Figure S7) at the largest fields that we apply, *E*_max_ = ±3.5 MV/cm. Comparing this spatially averaged switching remanent *E*-field to the polarization *P* determined by integrating the PUND switching current, we find that they have nearly identical dependences on the applied electric field (Figure 2f). The polarization *P* (measured by PUND) is plotted as a function of the applied field *E*_a_, which varies in a var PUND sequence between the peak var fields ± *E*_va_. The field  (measured by EBIC) is plotted as a function of the extremal set field ± |*E*_va_| of the preceding PUND measurement. During the EBIC image acquisitions the applied field *E*_a_ = 0. Averaged over the switching regions of the capacitor, the switching remanent field characteristic of a polarized domain is  MV/cm.

Because STEM EBIC imaging visualizes how much of the HZO is actually switching (24.5%, Figure S11), we can report both the polarization averaged over the whole capacitor (Figure 2e, up to 6.7 μC/cm^2^) and the polarization characteristic of the actively switching material (Figure 2f, up to 28 μC/cm^2^). This second value represents the 100% switching limit, and how closely this polarization limit is approached is typically not determined. Accordingly, this value is larger than (or at least comparable to) those reported for higher-quality films.^6,35,37,43,44^ Nonferroelectric regions constitute 39.4% of the capacitor area, with the remainder (35.9%) pinned in the *P*_↓_ state (Figure S11). Basically none of the capacitor is pinned in the *P*_↑_ state.

Combining the EBIC-informed PUND measurement of the polarization with the STEM EBIC measurement of the remanent field, we calculate the effective dead layer thickness as *d*/ϵ_rel-nf_ = 0.007 nm (eq S22). This value is encouragingly reasonable: making the same assumption about the dielectric constant (ϵ_nf_ = ϵ_fe_/2) as ref (2), we find the same value (*d* = 0.2 nm) for the thickness of the dead layer.

**Figure 3:**
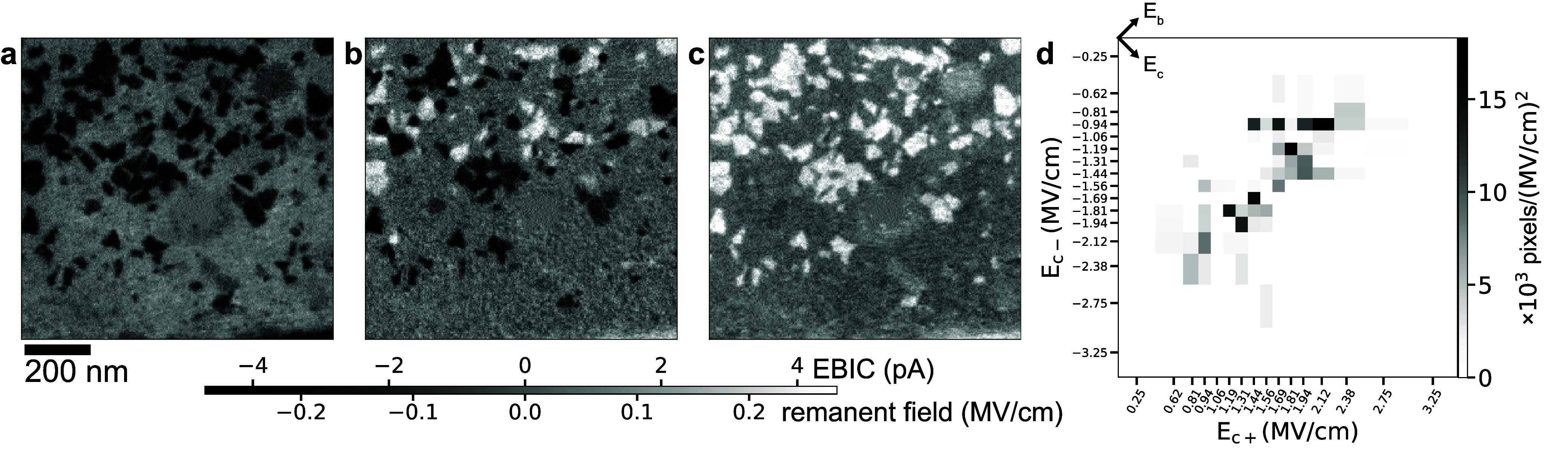
Domain-scale coercive field mapping. (a–c) STEM EBIC images of the region boxed in green in Figure 2, after PUNDp7, PUNDp3.5, and PUNDp0 pulse sequences, respectively. Here the same constant (i.e., nonswitching) background has been subtracted from each image, so (a) and (c) are showing the switching portion of ⟨*E*_r_⟩ for the maximal *P*_↑_ and *P*_↓_ states, respectively. Unlike that of Figure 2, this field of view shows no regions where the contrast is dominated by SEEBIC, so the intensity gray scale is given in both EBIC units (pA) and field units (MV/cm, Figure S3). (d) A 2D histogram of the positive and negative coercive fields *E*_*c*±_ for each pixel that switches at one, and only one, *E*_*c*+_ and *E*_*c*–_ (Figure S13).

To measure the coercive fields *E*_*c*±_ at the individual domain level, we repeat the experiment of Figure 2, collecting 34 PUND*+*EBIC data points, but this time imaging a 974 nm × 974 nm field of view (green box in Figures 2a–d, Movie M2). This region, only 4% of the full capacitor, is not necessarily representative, so quantitative agreement between the STEM EBIC measurements and the PUND measurements is no longer expected. Figure 3 shows three example images, corresponding to a maximal *P*_↑_ state (Figure 3a), a nearly depolarized (on average) state (Figure 3b), and a maximal *P*_↓_ state (Figure 3c), collected after PUNDp7, PUNDp3.5, and PUNDp0 pulse sequences, respectively. The nonswitching background, defined here to be the image [PUNDp7+NDPUn7+PUNDp0+NDPUn0]/4, has been subtracted from each image.

Subtracting, for instance, the PUNDp3 image from the PUNDp3.25 image ideally shows the regions that switch from *P*_↓_ to *P*_↑_ between *E*_*c*_ = 1.625 MV/cm and 1.5 MV/cm. We identify these regions via thresholding (Figure S12) and designate them as having *E*_*c*+_ = 1.56 MV/cm. (See Movies M3–M4 for a different approach.) Not every region switches with complete consistency. About 16% of the total area shows more than one *E*_*c*_ in one polarity, and about 36% shows an *E*_*c*+_ and not an *E*_*c*–_ in the data set, or vice versa (Figure S13). We confine our attention to the 57% that shows one *E*_*c*+_ and one *E*_*c*–_. Constructing a 2D histogram of the pixel-sized (3.8 nm × 3.8 nm) regions in this category (Figure 3d), we see a clear anticorrelation between these two variables; regions with large |*E*_*c*±_| tend to show small |*E*_*c*∓_|.

## Discussion

The switching current *I*(*E*_a_) (Figure 2e, red) measures the distribution of coercive fields *E*_*c*±_ averaged over the whole capacitor. Material inhomogeneities, variations in domain size, and/or grain misorientations, for instance, produce domain-to-domain variation in *E*_*c*±_. Because scaling reduces the number of domains per device, highly scaled ferroelectric field-effect transistors (FeFETs) benefit less from averaging and are more vulnerable to variations in threshold switching voltage — variations that might be unacceptable.^6,7^ Understanding the origins of the dispersion is thus a key step toward eventually enabling practical ferroelectric-based memory technologies.

**Figure 4:**
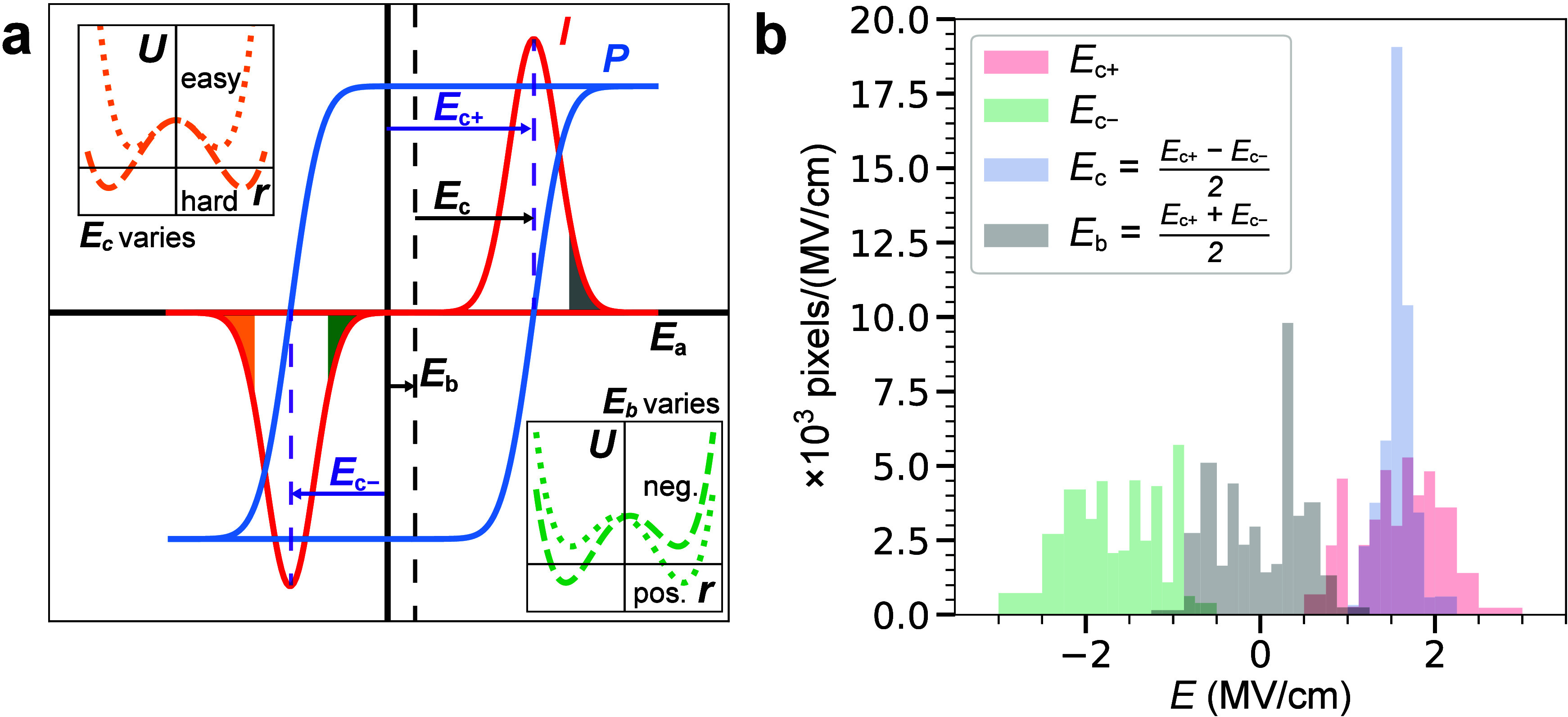
The origin of the dispersion in the *E*_*c*±_. (a) An ideal *P*(*E*) loop is centered, with coercive fields *E*_*c*±_ = ± *E*_*c*_ and a bias field *E*_*b*_ = 0. The part of the ferroelectric domain distribution that requires particularly large positive-*E* to switch is indicated in gray. It is not obvious whether this domain population switches back at a negative *E* that has a large (orange) or a small (green) magnitude. Insets show the potential energy *U* as a function of the atomic coordinate *r*(1,10) of the corresponding “easy/hard” model (orange) and “positive/negative” (green) model, respectively. (b) Projecting the Figure 3d 2D histogram onto its native 1D *E*_*c*±_ axes, and onto similar axes rotated by 45° in the *E*_*c*±_ plane,^48^ shows that the “positive/negative” hypothesis (green) is strongly favored. While the global *E*_*c*±_ can be measured with transport, the linear combinations (*E*_*c*+_ ± *E*_*c*–_)/2 cannot be ascribed to individual domains without imaging.

To provide a phenomenological explanation for the observed coercive field distributions, we consider two basic models (Figure 4). In the first model, some domains switch at small |*E*_a_| (“easy”) and others switch at large |*E*_a_| (“hard”). Various microscopic mechanisms might lead to this sorting. For instance, according to the “random bond” hypothesis,^10,45,46^ atomic defects at the level of the crystallographic unit cell could change the height of the potential barrier separating the two polarization states, creating domains with a range of ferroelectric coercivities (low and high corresponding to soft and hard, respectively). Alternatively, the degree of alignment between the capacitor’s *E*-field and the polar axis of the unit cell could vary,^35,47^ with parallel alignment giving easy switching and perpendicular alignment making switching difficult, if not impossible.

In the second model, some domains are biased toward positive polarization and others are biased toward negative polarization. This categorization relates to the “random field” model,^10,45,46^ where local defect structure introduces a bias one way or the other. It also captures imprint effects, where a domain’s prolonged soak in one polarization makes it harder to switch to the other.

Imprint effects are particularly noticeable after var pulses that are near *E*_*c*_; these pulses leave the capacitor in a mixed polarization state for the 6–10 min required to switch to EBIC imaging, acquire an EBIC image, and switch back to PUND. The subsequent PUND (or NDPU) init pulses show, via the ferroelectric switching currents, *E*_*c*±_ distributions that are strongly bimodal (Movie M1). Once the bimodal distribution has been established, it is not random: the same domains appear consistently in the early or the late part of the distribution, as the case may be. At present it is not clear whether this nonrandom behavior is initialized by “random” fields, or whether it can be ascribed to some more definite mechanism (*e.g.,* the local concentration of charged defects such as oxygen vacancies).

The evident anticorrelation between *E*_*c*±_ (Figure 3d) indicates that the positive/negative effect is the dominant source of dispersion. We define^48^ the coercive field *E*_*c*_ and the bias field *E*_*b*_ with
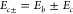
2The coercive field *E*_*c*_ is positive and is representative of the depth of the double-well potential (Figure 4a, left inset). The bias field *E*_*b*_ might be positive or negative, and it reflects the depth difference between the two sides of the double well (Figure 4a, right inset). Both vary from place to place in a real device because of inhomogeneities in, say, local grain orientation (*E*_*c*_), local charge distribution (*E*_*b*_), or defect concentrations (both). Projecting the 2D *E*_*c*±_ histogram onto four 1D axes, we find *E*_*c*+_ = 1.6 ± 0.5, *E*_*c*–_ = −1.5 ± 0.5, *E*_*c*_ = 1.6 ± 0.2, and *E*_*b*_ = 0.1 ± 0.5, all in MV/cm. The spread in the mean magnitude of the coercive field is 3× smaller than the other three; the coercive fields *E*_*c*_ are strikingly homogeneous once the effects of the local bias fields *E*_*b*_ are accounted for.

The *E*_*c*±_, *E*_*b*_, and *E*_*c*_ can be mapped on both the domain (Figure S14) and the device scale (Figure S16). At the domain scale this display format quantifies a fact that is obvious in the raw data (Movies M1–M2): many pixels switch together as discrete domains. The domain size is not obviously correlated with any of the coercive fields. While individual domains are only ≲20 pixels in the device-scale data set (Figure S16), they show the same anticorrelation between *E*_*c*+_ and *E*_*c*–_.

Not only can the nature of the dispersion in the coercive fields be identified, its origins can also be understood. The anticorrelation between *E*_*c*+_ and *E*_*c*–_ indicates that bias fields *E*_*b*_ play a significant determinative role in switching. STEM EBIC can map and measure background *E*-fields in the sample directly, and we expect these background fields to bias the coercive fields. Again focusing our attention on the domains that switch once and only once for each polarity (Movie M3), we curve fit to determine the remanent switchable fields  and remanent background fields  for each domain (Movie M4). (Movie M5 shows similar fitting, but pixel-by-pixel instead of domain-by-domain.) Plotting  versus the *E*_*b*_, we find another anticorrelation (S18–S19): domains with coercive fields that are biased negatively tend to have more positive background fields. Such a relationship is to be expected, because a polarity switch is generated by the sum of the applied field and the built-in background field. The correlation is less than one-to-one, but this is also to be expected.  is measured at *V* = 0 and is thus suppressed by screening (Figure S4). *E*_*b*_ is determined by the *E*_*c*±_, which occur at *V* ≠ 0 that removes the screening to increase the depolarization field. Thus, the bias fields, whose existence we inferred by determining when each domain switches, are evident in the remanent domain fields that do *not* switch.

## Conclusion

The STEM EBIC generated in a capacitor is linear in the applied voltage *V*. This simple relationship allows for an equally simple and quantitative interpretation: STEM EBIC images are *E*-field maps. Because the contrast generation mechanism is independent of magnification, STEM EBIC imaging provides unparalleled flexibility in choosing the field of view: it is able both to quickly survey an entire device and to detail individual domains. In the absence of an applied field, STEM EBIC imaging maps the remanent fields ⟨*E*_r_⟩. Polarization reversals are obvious in the raw data. By successively imaging a ferroelectric capacitor subsequent to polarizing voltage pulses of varying magnitudes, the signed coercive fields *E*_*c*±_ for each ferroelectric domain can be determined quantitatively. Applied to a polycrystalline sample of ALD HZO, STEM EBIC imaging reveals that the coercive field *E*_*c*_ varies little from domain to domain, and that most of the dispersion in the *E*_*c*±_ = *E*_*b*_ ± *E*_*c*_ is the result of the locally varying offset bias fields *E*_*b*_. Moreover, the remanent fields ⟨*E*_r↓↑_⟩ = ⟨*E*_rb_⟩ ∓ ⟨*E*_rs_⟩ measured with STEM EBIC can be decomposed into the nonswitching background ⟨*E*_rb_⟩ and the switching field ⟨*E*_rs_⟩. The background fields ⟨*E*_rb_⟩ shift the coercive field biases *E*_*b*_ in the expected manner. Thus, not only can STEM EBIC imaging determine the bias fields *E*_*b*_ for each domain, it can also map the nonswitching fields ⟨*E*_rb_⟩ that produce that bias. By providing such a straightforward, multifaceted, and quantitative picture of the key properties of a ferroelectric film, STEM EBIC imaging promises insights to aid in the development of reliable and high-performing ferroelectric materials.

## Methods

### Fabrication

We characterize a 30/20/20 nm TaN/HZO/TaN capacitor (Figure 1) fabricated on a 20 nm-thick Si_3_N_4_ membrane window. The window is supported by a 200-μm-thick silicon substrate that serves as the window’s frame. The electrodes are patterned in three separate rounds of lithography. First, 5/25 nm Ti/Pt electrodes are patterned with optical lithography and deposited via e-beam evaporation.^31^ These contact the bottom electrode (BE, 30 nm thick) and top electrode (TE, 20 nm thick), which are tantalum nitride deposited in separate rounds of e-beam lithography via DC magnetron sputtering from a sintered TaN target. The capacitor’s TE and BE are square with area 4.7 μm × 4.7 μm, with rotation of 5° between them such that the effective capacitor area *A* = 22 μm^2^.

The ferroelectric Hf_0.5_Zr_0.5_O_2_ (HZO) layer between the TE and BE has thickness  nm and is deposited via plasma-enhanced atomic layer deposition (PE-ALD).^43,44^ Briefly, the PE-ALD is performed within an Oxford FlexAL II instrument with a table temperature of 260°C. Tetrakis(ethylmethyl)amido hafnium and tetrakis(ethylmethyl)amido zirconium serve as the hafnium and zirconium precursors. An oxygen plasma generated from an inductively coupled plasma source is used as the oxygen precursor. Films are prepared with 17 supercycles containing a 6:4 ratio of hafnium and zirconium, resulting in an approximate composition of Hf_0.5_Zr_0.5_O_2_. In the final fabrication step, the HZO, which is amorphous as deposited, is crystallized with a rapid thermal anneal (RTA) at 700°C in nitrogen within an Allwin21 AccuThermo 610 RTA instrument. The capacitor’s linear, nonhysteretic capacitance is about 500 fF with a dielectric constant of 50ϵ_0_. Its DC resistance at ±1 V is greater than 600 GΩ.

### Transport

In the PUND technique,^40^ two pairs of identical voltage pulses (positive and negative) are applied to the sample. The first pulse produces switching and nonswitching currents, while the second produces only nonswitching currents. The nonswitching currents are primarily due to the linear response of the capacitor and stray capacitance in the circuit. Subtracting the second current pulse from the first gives the quantity of interest: the capacitor’s (ferroelectric) switching current as a function of applied voltage. We use an extension of the PUND technique, termed “the nano-PUND”,^41^ that is particularly well suited for small capacitors.

Channel 1 of a Rigol DG2102 arbitrary function generator sources triangular PUND voltage pulses to the capacitor’s BE at a rate of 25 × 10^6^ samples/s with |*dV*/*dt*| = 28 kV/s. Between each triangular pulse there is a 7.5 ms delay (Figure S1). To generate the nano-PUND currents that cancel the currents arising from stray capacitance in the circuit of interest, a 21 pF auxiliary capacitor is simultaneously driven with a similar waveform produced by Channel 2 of the Rigol. An NF CA5351 TIA (NF Corp.) connected to a National Instruments USB-5133 digitizer captures the PUND current response from the TE at 25 × 10^6^ samples/s.

To provide initialization and additional diagnostic capability, we use extended PUND sequences that first wipe the previous polarization (“init”), and then measure (“var”) and program (“set”) a new one (Figure S1). The init pulses are two full ±7 V PUND waveforms. The var and set pulses together are two-and-a-half PUND waveforms at the variable voltage. Sequences of the opposite polarity (NDPU) are also used. The triangle waveforms’ voltage ramp rate magnitude |d*V*/d*t*| is held constant at 28 kV/s (7 V in 250 μs), so the frequency of the var and set waveforms is generally higher than that of the init waveform. Between each triangular pulse is a delay of 7.5 ms. A full waveform thus consists of PUNDPUNDpundpundpu (PUNDp) or NDPUNDPUndpundpund (NDPUn), where capital letters indicate triangular pulses with peak magnitude of 7 V and lower case letters indicate triangular pulses with a peak magnitude in the range 0–7 V. A number appended to PUNDp or NDPUn indicates the variable (var and set) magnitude. Thus, nominally PUNDp7 leaves the capacitor in its maximal remanent *P*_↑_ state, while PUNDp0 leaves it in its maximal remanent *P*_↓_ state, since the last peak voltage seen by the capacitor is +7 V and −7 V, respectively.

The PUND data is internally consistent: the polarization curve generated by piecing together measurements over the whole 40-point data set (blue triangles in Figure 2f) tracks the polarization curve generated in the first PUNDp7 sequence (blue curve). (Every PUNDp and NDPUn sequence contains two *P*(*E*) measurements over the full *E*_a_ = ±3.5 MV/cm range.) While imprint effects produced by the capacitor’s immediate prehistory are noticeable, there are no signs of wake-up or fatigue over the course of the experiment. STEM EBIC imaging dozens of times does no discernible damage to the capacitor’s ferroelectric response.

### Microscopy

STEM EBIC imaging is performed on a G1 FEI Titan 80–300 operated with an acceleration voltage of 300 kV, a 50 μm C2 aperture, a 10 mrad convergence semiangle, and a 300 mm camera length. The beam current measured at the fluorescent screen is 150 pA for the data of Figure 2 and 170 pA for the data of Figures 3 and S8. The BF and ADF collection half-angles are 0–2 mrad and 25–154 mrad, respectively. Images are 256 × 256 pixels with a 2.5 ms/pixel dwell time and a frame time of 197 s, excepting the calibration data (Figure S3) images, which are 128 × 128 with a frame time of 49 s, and the data of Figure S8. The Figure 2 images have 21.5 nm pixels with a 5.51 μm field of view. The Figure 3 images have 3.81 nm pixels with a 974 nm field of view. EBICs were collected using NEI’s EBIC system (NanoElectronic Imaging, Inc.). The system, which includes a TEM sample holder manufactured by Hummingbird Scientific, was used independently and with external NF SA-608F2 amplifiers. All experiments were performed at room temperature in the microscope’s high vacuum.

We quantify the magnitude of the *E*-field that generates the EBIC contrast with the aid of a separate calibration measurement (Figure S3). By applying a voltage *V* to the BE TIA’s common relative to the TE TIA’s common, we offset the potential of the BE, which is at the “virtual ground” of the BE TIA. This offset produces an  inside the capacitor, where  is the HZO thickness (Figure S4). EBIC imaging the capacitor while it supports this known *E*-field provides the desired relationship between a measured EBIC and the contrast-generating *E*-field. The relationship is gratifyingly linear for fields ≤0.5 MV/cm (Figure S3), which are large in comparison to the remanent fields observed (typically ≲0.3 MV/cm).

To the extent that electron–hole pair separation is the only source of EBIC, the top- and bottom-electrode TIAs produce EBIC images with equal and opposite contrast. Taking “the” EBIC to be (*I*_top_ – *I*_bot_)/2 doubles the magnitude of the signal of interest in comparison to signals producing currents in only one TIA. Thus, signals resulting from secondary electron (SE) generation^31^ or beam absorption, which are already small, are further suppressed. The resulting EBIC image (e.g., Figure 2a,b) highlights regions with built-in electric fields, so ferroelectric domains appear with bright or dark contrast, depending on whether the contrast-generating *E*-field is positive (directed up) or negative (directed down), respectively. Here the use of two EBIC TIAs, one for the TE and one for the BE, provides an invaluable experimental handle. That hysteretic changes appear in the difference images and not in the sum images confirms that these effects are due to electric fields (Movies M1–M2, Figures S5–S6).

It is not obvious *a priori* whether, say, *P*_↑_ will produce positive or negative EBIC contrast. In the absence of an applied voltage, both the TE and the BE are at virtual ground during an EBIC measurement. Thus, *V* = −∫*E*·*dz* = 0 when the range of integration extends from one electrode to the other (Figure S4). Correspondingly, for *P*_↑_ there must be fields *E*_nf_ > 0 in the nonferroelectric (dead) layers at the electrodes to compensate the depolarization field *E*_fe_ < 0 in the bulk HZO.^2,11,49−53^ Presumably the dead layer thickness , so |*E*_nf_| ≫ |*E*_fe_|. Our measurements indicate that the EBIC-contrast-generating *E*-field ⟨*E*_r_⟩ is the bulk field *E*_fe_: we measure EBIC_*P*_↑__ < EBIC_*P*_↓__. (When reporting *E*-field measurements performed with EBIC, we use angle brackets ⟨*E*⟩ to indicate that all such measurements represent a *z*-column average over the full thickness of the sample. We use an overbar  to indicate an additional, multipixel *xy* spatial average. See the SI for an extended description of our sign determinations, vocabulary, notation, and contrast model.)

## Data Availability

All data needed to evaluate the conclusions in the paper are present in the paper and/or the SI. The raw data are available on Dryad.

## Supporting Information Available

Detailed theoretical analysis, movie captions, and Figures S1–S20 with captions (PDF)
Device-scale imaging of polarization switching (MP4)
Domain-scale imaging of polarization switching (MP4)
Remanent field ⟨*E*_r_⟩ evolution with set voltage *V* (MP4)
Domain-by-domain switching behavior (MP4)
Pixel-by-pixel switching behavior (MP4)
