# Supplementary material for: Mapping Ferroelectric Fields Reveals the Origins of the Coercivity Distribution
Source: ACS Nano. 2024 Jul 17;18(31):20380–8. doi: 10.1021/acsnano.4c04526 (PMC11308779; doi:10.1021/acsnano.4c04526)
Supplement: Supplementary file 1 — nn4c04526_si_001.pdf [file nn4c04526_si_001.pdf]

Supporting Information for:

Mapping Ferroelectric Fields Reveals the Origins of the Coercivity Distribution

Ho Leung Chan[1,2], Shelby S. Fields[3], Yueyun Chen[1,2], Tristan P. O'Neill[1,2], Megan K. Lenox[3], William A. Hubbard[4], Jon F. Ihlefeld[3,5], and Brian C. Regan[1,2,4*]

[1]*Department of Physics and Astronomy, University of California, Los Angeles, CA 90095, U.S.A*
[2]*California NanoSystems Institute, University of California, Los Angeles, CA 90095, U.S.A*
[3]*Department of Materials Science and Engineering, University of Virginia, Charlottesville, VA 22904, U.S.A.*
[4]*NanoElectronic Imaging, Inc., Los Angeles, CA 90095, U.S.A.*
[5]*Charles L. Brown Department of Electrical and Computer Engineering, University of Virginia, Charlottesville, VA 22904, U.S.A.*

Contents

SI.1 Signs . . . **2**
SI.2 Vocabulary of PUND . . . **3**
SI.3 STEM EBIC in a ferroelectric capacitor . . . **4**
SI.4 Supplementary Movies . . . **6**
SI.5 Supplementary Figures . . . **11**

Figures

M1 Device-scale imaging of polarization switching . . . 6
M2 Domain-scale imaging of polarization switching . . . 7
M3 Remanent field $\langle E_{\rm r}\rangle$ evolution with set voltage V. . . . 8
M4 Domain-by-domain switching behavior . . . 9
M5 Pixel-by-pixel switching behavior . . . 10
S1 PUND sequence . . . 11
S2 PUND-derived $P(E)$ loops. . . . 12
S3 EBIC vs. E-field calibration . . . 13
S4 Field geometry and contrast model . . . 14
S5 Whole-device imaging of $P_{\updownarrow}$ states with BF, ADF, and STEM EBIC . . . 15
S6 Domain-scale imaging of $P_{\updownarrow}$ states with BF, ADF, and STEM EBIC . . . 16
S7 Device-scale capacitor and switching masks . . . 17
S8 Device-scale high-resolution EBIC images . . . 18
S9 Device-scale high-resolution $\langle E_{\rm r}\rangle$ distributions . . . 19
S10 Model of background field $\langle E_{\rm rb}\rangle$ effects on switching . . . 20
S11 Device-scale switching map . . . 21
S12 Identifying switching domains . . . 22
S13 Domain-scale switch count . . . 23
S14 Domain-scale $E_{c\pm}$, E_b, and E_c maps . . . 24
S15 Domain-scale $\overline{\langle E_{\rm rb}\rangle}$, $\overline{\langle E_{\rm rs}\rangle}$, E_b, and E_c maps . . . 25
S16 Device scale $E_{\pm}$ maps and distribution . . . 26
S17 Shift in $\overline{\langle E_{\rm r}\rangle}$ for $P_{\uparrow}$ and $P_{\downarrow}$ separately . . . 27
S18 Local background fields shift $E_{c\pm}$, domain scale . . . 28
S19 Local background fields shift $E_{c\pm}$, device scale . . . 29
S20 Domain-scale $\langle E_{\rm rb}\rangle$, $\langle E_{\rm rs}\rangle$, E_b, and E_c maps, pixel-by-pixel . . . 30

*Corresponding author: regan@physics.ucla.edu

SI

SI.1 Signs

In STEM EBIC imaging, unlike SEM EBIC imaging, the beam is not absorbed by the sample. Because this otherwise troublesome background is absent, smaller signals are detectable. In particular, secondary-electron electron beam-induced current (SEEBIC) can make a detectable contribution to the image contrast.[31]

We use this contribution to verify the polarity of our electrical connections, which provides a valuable check against sign errors. We also collect EBICs from opposing electrodes using two transimpedance amplifiers (TIAs) simultaneously. With two TIAs, we capture both the electron and the hole from every electron-hole pair separated. We thus can distinguish non-E-field contrast from the electron-hole pair separation contrast characteristic of built-in E-fields.

A necessary precondition for distinguishing these contrast mechanisms is sub-pA EBIC sensitivity. In these experiments, unlike more traditional applications of EBIC imaging, the E-field-generated EBICs are < 10 pA and thus not orders-of-magnitude larger than the non-E-field-generated EBICs. We use TIAs with a front-end transimpedance gain of 100 GΩ, which puts the shot noise in 1 pA above the Johnson noise floor and makes the signals generated by both contrast mechanisms well-resolved.

On the boundaries of the low-magnification images (*e.g.,* Fig. 1, Figs. 2a, b, and d), away from the capacitor body, we can identify, according to the materials present, three types of region: HZO with Si_3N_4, TE with HZO and Si_3N_4, and BE with HZO and Si_3N_4. These latter two regions allow us to unambiguously identify the polarity of the electrical connections used to acquire a given EBIC image.[31] Because secondary electron emission leaves holes behind, when the beam is incident on, say, the TE, the TIA connected to the TE detects a positive (hole) current that appears in the image as bright contrast. Recapture of secondary electrons from the TE produces a negative (electron) current in the BE. This EBIC is measured by the TIA connected to the BE and appears as dark contrast in the corresponding image. Thus the TE (BE) appears bright (dark) in the image generated by the TIA connected to the TE, and vice versa. For instance, in Fig. 1 the TE connection is made at the bottom of the image and the BE connection is made at the top. In Figs. 2a–c, which include non-ferroelectric contributions to $(I_{\mathrm{top}} - I_{\mathrm{bot}})/2$, the connection at the bottom (top) of the image is correspondingly bright (dark). Thus SEEBIC contrast in the low-magnification images allows us to verify our signal polarities in the data, independent of our notebook record of the experiment. The simultaneous use of two TIAs, as opposed to just one, provides an additional check.

We list our sign conventions here. When performing PUND or calibrating the EBIC vs. E-field relationship (Fig. S3), we apply the voltage V to the BE relative to the TE, which is defined to be "ground". A positive voltage then produces a positive E-field (*i.e.,* one directed upwards). During, say, calibration, electron-hole pairs produced by the beam are separated in this field, with a positive (hole) current going to the TE and the negative (electron) current going to the BE. We display the EBIC as $I_{\mathrm{EBIC}} \equiv (I_{\mathrm{top}} - I_{\mathrm{bot}})/2$ with bright contrast indicating positive values. (The EBIC produced by electron-hole pair generation flows with complete uniformity from one transimpedance amplifier through the capacitor to the other transimpedance amplifier.) Thus our EBIC images show the E-field (not the polarization): positive (bright) contrast indicates a positive (up) E-field in the capacitor. PUNDp waveforms with sufficiently large positive set voltages (PUNDp7 being the best example) produce 'up' polarization ($P_\uparrow$). Once the applied voltage is returned to zero, the remanent polarization is still positive, but the remanent, depolarizing E-field in the bulk of the dielectric (E_{fe}) is negative (Fig. S4). The line integral $\int_{\mathrm{BE}}^{\mathrm{TE}} E \cdot dz$ (Eq. S5) is zero, however, so at each dielectric-electrode interface there is a non-ferroelectric compensation layer containing a positive E-field (E_{nf}).[49] To the extent that the compensation layers are thin ($d \ll \ell$) in comparison to the thickness of the ferroelectric, $|E_{\mathrm{nf}}| \gg |E_{\mathrm{fe}}|$.

Every statement in the previous paragraph is either a description of our conventions or standard electromagnetic theory. The following statement is neither: subtracting a STEM EBIC image acquired after NDPUn7 (*e.g.,* Fig. 2b) from one acquired after PUNDp7 (*e.g.,* Fig. 2a), we find the changed regions to have $I_{\mathrm{EBIC}} < 0$ (*e.g.,* Fig. 2d). We thus conclude that the STEM EBIC contrast is generated by the remanent depolarization field E_{fe} in the bulk and not by the screening fields E_{nf} at the bulk/electrode interfaces.

The previous EBIC study[29] of ferroelectric HZO comes to the opposite conclusion. We note, however, that the contrast mechanisms of SEM EBIC are more complicated than those of STEM EBIC, with non-negligible EBIC contributions from regions merely neighboring the beam position.[29] Moreover, the signs in Ref. 29 are not internally consistent. Because SEEBIC contrast[31] provides a signal of known polarity in our raw data and because we collect EBICs from both the TE and the BE, it is relatively easier to guard against sign errors.

A few signs in the analysis are tricky enough to warrant special mention. For instance, because $E_{\mathrm{fe}} \simeq \langle E_{\mathrm{rs}} \rangle$ is opposite the polarization P (Fig. S4), the y-axes for Fig. 2f have opposite orientations. Also, $\langle E_{\mathrm{rb}} \rangle$ and E_b are expected to have opposite signs. Consider an imprint field that develops for the $P_\uparrow$ state diagrammed in Fig. S4, which will be positive and will reduce the depolarizing field E_{fe}. This imprint field makes it more difficult to switch to the $P_\downarrow$ state; more negative voltages are required, which implies a negative bias field E_b.

SI.2 Vocabulary of PUND

The polarization $\mathbf{P}$ is related to the electric displacement $\mathbf{D}$ and the electric field $\mathbf{E}$ by

$$\mathbf{D} \equiv \epsilon_0 \mathbf{E} + \mathbf{P}. \tag{S1}$$

Following the standard practice, we describe our PUND measurements as measurements of the polarization P. This language is not completely accurate,[50] as we explain below.

Our transport measurement equipment only sees the free currents $I_{\rm free} = \int \mathbf{J}_{\rm free} \cdot d\mathbf{a}$, where the current density $\mathbf{J}_{\rm free}$ obeys the continuity equation

$$\nabla \cdot \mathbf{J}_{\rm free} + \frac{\partial \rho_{\rm free}}{\partial t} = 0. \tag{S2}$$

The free charge density $\rho_{\rm free}$ is related to the displacement field $\mathbf{D}$ by the first Maxwell equation,

$$\nabla \cdot \mathbf{D} = \rho_{\rm free}, \tag{S3}$$

which relates $\rho_{\rm free}$ to the polarization *via* Eq. S1 and

$$\rho \equiv \rho_{\rm free} + \rho_{\rm b}, \tag{S4}$$

where ρ is the total charge density and the bound charge density is defined by $\rho_b \equiv -\nabla \cdot \mathbf{P}$.

Specializing to our particular geometry (Fig. S4), we allow for a potential V between the TE and the BE,

$$V = -\int_{\rm TE}^{\rm BE} E \cdot dz, \tag{S5}$$

where the integration limits account for the fact that the voltage V is applied to the bottom electrode with the top electrode defined to be at $V = 0$. For a ferroelectric capacitor (Fig. S4), we have:

$$V = E_{\rm fe}\, \ell + 2E_{\rm nf}\, d, \quad \text{and} \tag{S6}$$

$$D = \sigma_{\rm free} = \epsilon_{\rm nf} E_{\rm nf} = \epsilon_0 E_{\rm fe} + P_{\rm fe}. \tag{S7}$$

Here $E_{\rm fe}$ is the electric field in the ferroelectric and $E_{\rm nf}$ is the electric field in the non-ferroelectric layers at the ferroelectric-electrode interfaces. We do not distinguish between non-ferroelectric layers that are "dead" or "passive" and those that are screening. In other words, these layers might be in the dielectric or in the metal — the analysis that follows applies in both cases. Equation S6 follows from the definition of voltage (Eq. S5). Equation S7 follows from the first Maxwell equation (Eq. S3), which says that D is continuous inside the capacitor, where $\rho_{\rm free} = 0$.

The ferroelectric polarization $P_{\rm fe} = \sigma_{\rm b}$ ($\sigma_{\rm b}$ is the bound surface charge density) includes both hysteretic and non-hysteretic contributions. It is not directly measurable with transport if the dead layer thickness d is non-zero. Eliminating $E_{\rm fe}$ and $E_{\rm nf}$ from Eqs. S6–S7, we find the free charge[49,50]

$$\sigma_{\rm free} = \frac{P_{\rm fe} + \frac{\epsilon_0}{\ell} V}{1 + \frac{2d}{\ell}\frac{\epsilon_0}{\epsilon_{\rm nf}}}. \tag{S8}$$

Transport measures the free current,

$$\frac{d\sigma_{\rm free}}{dt} = \frac{\frac{dP_{\rm fe}}{dt} + \frac{\epsilon_0}{\ell}\frac{dV}{dt}}{1 + \frac{2d}{\ell}\frac{\epsilon_0}{\epsilon_{\rm nf}}}. \tag{S9}$$

In PUND, the "U" pulse is subtracted from the "P" pulse. (The "N" and "D" pulses are handled similarly.) The current density plotted is thus

$$\Delta\left(\frac{d\sigma_{\rm free}}{dt}\right) \equiv \left.\frac{d\sigma_{\rm free}}{dt}\right|_{\text{“}P\text{”}} - \left.\frac{d\sigma_{\rm free}}{dt}\right|_{\text{“}U\text{”}} = \frac{\frac{dP_{\rm fe}}{dt}\big|_{\text{“}P\text{”}} - \frac{dP_{\rm fe}}{dt}\big|_{\text{“}U\text{”}}}{1 + \frac{2d}{\ell}\frac{\epsilon_0}{\epsilon_{\rm nf}}}. \tag{S10}$$

The "polarization"[50] is found by time-integrating this equation, which gives

$$\Delta D = \Delta\sigma_{\rm free} \equiv \int_{\text{“}P\text{”}} \frac{d\sigma_{\rm free}}{dt} dt - \int_{\text{“}U\text{”}} \frac{d\sigma_{\rm free}}{dt} dt = \frac{\Delta P_{\rm fe}}{1 + \frac{2d}{\ell}\frac{\epsilon_0}{\epsilon_{\rm nf}}}. \tag{S11}$$

This expression describes what PUND measures over a half-cycle (*i.e.,* over "PU"). Because $\Delta D \neq \Delta P_{\rm fe}$, it is not completely accurate to say that PUND measures the ferroelectric polarization $P_{\rm fe}$. PUND measures $\Delta D = \Delta\sigma_{\rm free}$, as given by Eq. S11. The approximation that PUND measures $\Delta D = \Delta\sigma_{\rm free} \simeq \Delta P_{\rm fe}$ has historically been quite good,

but as the thickness ℓ of ferroelectric films approaches the atomic scale the quantity $\frac{2d}{\ell}\frac{\epsilon_0}{\epsilon_{\rm nf}}$ is no longer necessarily negligible.

To avoid slowing our development with these fine distinctions, we skate over the inaccuracies inherent in the "polarization" language[50] in the main text. (For instance, the "current density" plotted in Fig. 2e is $\frac{d}{dt}\Delta\sigma_{\rm free}$.) However, an awareness of such details is helpful in understanding our STEM EBIC contrast, to be described in the following section.

We note that we also follow the standard practice of reporting the applied electric fields (*e.g.,* $E_{\rm a}$ and $E_{\rm va}$) as V/ℓ, where V is the applied voltage and ℓ is the nominal film thickness. Voltages are unambiguous, but, for comparisons between different devices, electric fields are preferred since the fields are more device-independent. While it would be more correct to take the E-fields to be $V/(\ell+2d)$, the thickness d of the non-ferroelectric layers is not well-known. This error ($\lesssim 4\%$) carries over into our reported values for the coercive fields ($E_{c\pm}, E_c, E_b$).

Some authors decompose ferroelectric polarization into two components,[50]

$$\mathbf{P}_{\rm fe} = \epsilon_0\chi_{\rm fe}\,\mathbf{E}_{\rm fe} + \mathbf{P}_{\rm d}, \tag{S12}$$

which is to say, into a part that is linear in the electric field $\mathbf{E}$ and a part that is due to "the switching dipoles". The distinction between $\mathbf{P}_{\rm fe}$ and $\mathbf{P}_{\rm d}$ is not always made clear (*e.g.,* Ref. 2). If necessary, this decomposition is best performed at the end of the analysis, not the beginning, because it confuses otherwise clear distinctions between linear and non-linear, on the one hand, and hysteretic and non-hysteretic on the other. Applying Eq. S1 and Eq. S12 to a ferroelectric we have

$$\mathbf{D} = \epsilon_0\mathbf{E}_{\rm fe} + \mathbf{P}_{\rm fe} = \epsilon_{\rm fe}\mathbf{E}_{\rm fe} + \mathbf{P}_{\rm d}, \tag{S13}$$

with $\epsilon_{\rm fe} = \epsilon_0(1+\chi_{\rm fe}) = \epsilon_0\epsilon_{\text{rel-fe}}$.[50]

SI.3 STEM EBIC in a ferroelectric capacitor

'Standard' EBIC (as opposed to SEEBIC) is generated when beam-induced electron-hole pairs get separated in local electric fields.[31] To model the standard EBIC contrast generated by the HZO, we consider the same idealized scenario treated in the previous section (Fig. S4). Starting again with Eqs. S6–S7, but this time solving for $E_{\rm fe}$ and $E_{\rm nf}$, we find[49,50]

$$E_{\rm fe} = \frac{\frac{V}{\ell} - \frac{2d}{\ell}\frac{P_{\rm fe}}{\epsilon_{\rm nf}}}{1+\frac{2d}{\ell}\frac{\epsilon_0}{\epsilon_{\rm nf}}} = \frac{V}{\ell} - \frac{2d}{\ell}\frac{\sigma_{\rm free}}{\epsilon_{\rm nf}} \quad \text{and} \tag{S14}$$

$$E_{\rm nf} = \frac{\epsilon_0\frac{V}{\ell} + P_{\rm fe}}{\epsilon_{\rm nf}\left(1+\frac{2d}{\ell}\frac{\epsilon_0}{\epsilon_{\rm nf}}\right)} = \frac{\sigma_{\rm free}}{\epsilon_{\rm nf}}. \tag{S15}$$

Thus, for $V=0$ as $d\to 0$, $\sigma_{\rm free}\to P_{\rm fe}=\sigma_{\rm b}$, $E_{\rm fe}\to 0$, and $E_{\rm nf}\to P/\epsilon_{\rm nf} = \sigma_{\rm b}/\epsilon_{\rm nf}$. The case of perfect screening ($d=0$) leaves no field at all in the ferroelectric, but the dead layer field remains finite.

According to Eq. S14, the electric field in the bulk HZO $E_{\rm fe}$ is linear in V, $P_{\rm fe}=\sigma_{\rm b}$, and $\sigma_{\rm free}$. The calibration measurements (Fig. S3) demonstrate that the EBIC is linear in V. We also find that the measured EBIC has the sign expected for a current generated in the bulk of the HZO (Sec. SI.1). To interpret the measured EBIC simply, we thus assume that it is wholly generated in the bulk of the HZO. Any EBIC generated in the passive layer, which would tend to reduce the net EBIC, we assume is negligible.

To provide a notational reminder of these assumptions, we write the electric fields that we derive from EBIC measurements in angle brackets. For instance, when speaking of EBIC measurements of the remanent electric field, we write $\langle E_{\rm r}\rangle$. Because the sample is electron-transparent, the beam interacts with a relatively small area of the sample, measured in the transverse (x and y) directions. But the beam necessarily interacts with an entire z column as it passes through the sample. The remanent electric fields change magnitude and even sign along this column (Fig. S4), so it is appropriate to indicate the EBIC E-field measurements as returning a weighted average of the E-field along this column. Averaging over a small transverse area due to the beam's non-zero interaction volume is also implicit in the angle-bracket average. Overbars (*e.g.,* $\overline{\langle E_{\rm rs}\rangle}$) are used to indicate a different spatial average, namely a multi-pixel transverse (*i.e., xy*) average which might, for example, be over a particular domain or over all of the switching regions, as the case may be.

We can now use Eq. S1, which defines the displacement field $\mathbf{D}$, to summarize our experimental method:

$$\overbrace{\mathbf{D}}^{\text{PUND}} = \overbrace{\epsilon_0\mathbf{E}}^{\text{EBIC}} + \mathbf{P}. \tag{S16}$$

In words, PUND measures the displacement field $\mathbf{D}$ while STEM EBIC measures the electric field $\mathbf{E}$ inside the ferroelectric. Actually, STEM EBIC plays an auxiliary but essential role in determining $\mathbf{D}$ as well, in that it measures

the switching area A_s and thus enables the calculation of the ferroelectric current density from the currents $\Delta I_{\text{free}} = A_s \Delta d\sigma_{\text{free}}/dt$. The parts of the capacitor that do not switch are invisible to PUND. Thus, by visualizing the switching area A_s, STEM EBIC is directly determining the specific unknown required to convert I_{free} to $\mathbf{D}$ (which we can then relate to $\mathbf{E}$)[1].

To be more explicit, from Eq. S7 and Eq. S13 we have

$$\sigma_{\text{free}} = \epsilon_0 E_{\text{fe}} + P_{\text{fe}} = \epsilon_{\text{fe}} E_{\text{fe}} + P_{\text{d}}, \text{ so} \tag{S17}$$

$$\Delta\sigma_{\text{free}} = \epsilon_0 \, \Delta\langle E_{\text{r}}\rangle + \Delta P_{\text{fe}} = \epsilon_{\text{fe}} \, \Delta\langle E_{\text{r}}\rangle + \Delta P_{\text{d}} \tag{S18}$$

where we are now equating the electric field in the ferroelectric E_{fe} with the remanent electric field $\langle E_{\text{r}}\rangle$ measured with STEM EBIC. PUND measures the switching displacement field ΔD *via* the switching currents $\Delta\frac{d\sigma_{\text{free}}}{dt}$, which we integrate to find $\Delta\sigma_{\text{free}}$. STEM EBIC measures the electric field in the ferroelectric E_{fe}, particularly the part of the field that switches with the polarization direction, $\Delta\langle E_{\text{r}}\rangle = -2\langle E_{\text{rs}}\rangle$.

Dividing Eq. S18 by $\Delta\sigma_{\text{free}}$ gives

$$1 = -\epsilon_0 \frac{2\langle E_{\text{rs}}\rangle}{\Delta\sigma_{\text{free}}} + \frac{\Delta P_{\text{fe}}}{\Delta\sigma_{\text{free}}} = -\epsilon_{\text{fe}} \frac{2\langle E_{\text{rs}}\rangle}{\Delta\sigma_{\text{free}}} + \frac{\Delta P_{\text{d}}}{\Delta\sigma_{\text{free}}}. \tag{S19}$$

Using our measured (Fig. 2f) values $\Delta\sigma_{\text{free}}/2 = 28$ μC/cm^2, $\overline{\langle E_{\text{rs}}\rangle} = 0.23$ MV/cm, and $\epsilon_{\text{fe}} = 50\epsilon_0$ gives

$$1 = -7.3 \times 10^{-4} + \frac{\Delta P_{\text{fe}}}{\Delta\sigma_{\text{free}}} = -3.6 \times 10^{-2} + \frac{\Delta P_{\text{d}}}{\Delta\sigma_{\text{free}}}. \tag{S20}$$

Thus, the displacement field and the polarization are nearly equal inside the ferroelectric, while the electric field is of the opposite sign and is almost negligible in comparison. Screening reduces the uncompensated depolarization field E_{fe} by a factor of 27.

Knowing the screening factor, we can now calculate the thickness of the screening layers. According to Eq. S14 at $V = 0$,

$$E_{\text{fe}} = -\frac{2d}{\ell}\frac{\sigma_{\text{free}}}{\epsilon_{\text{nf}}} \tag{S21}$$

Thus it follows that

$$\frac{d}{\epsilon_{\text{rel-nf}}} = -\frac{\epsilon_0 \overline{\langle E_{\text{rs}}\rangle}}{\Delta\sigma_{\text{free}}}\ell = 0.007 \text{ nm}. \tag{S22}$$

The minus sign merely indicates that σ_{free} and E_{fe} have opposite signs (Fig. S4); d is a positive number. If we follow Ref. 2 by taking $\epsilon_{\text{nf}} = \epsilon_{\text{fe}}/2$ ($\epsilon_{\text{fe}} = 50\epsilon_0$ for our HZO films), we find $d = 0.2$ nm.

The estimate given in Eq. S22 might be a lower bound. We are assuming $E_{\text{fe}} = \langle E_{\text{r}}\rangle$. To the extent that some canceling EBIC is generated in the dead layers, the estimate of $|E_{\text{fe}}|$ is low and the estimate of $d/\epsilon_{\text{rel-nf}}$ is correspondingly small.

[1] The switching area A_s is determined by subtracting and thresholding STEM EBIC images (*e.g.,* Fig. S7d and Fig. S12b). This determination is independent of the E-field calibration (Fig. S3).

SI.4 Supplementary Movies

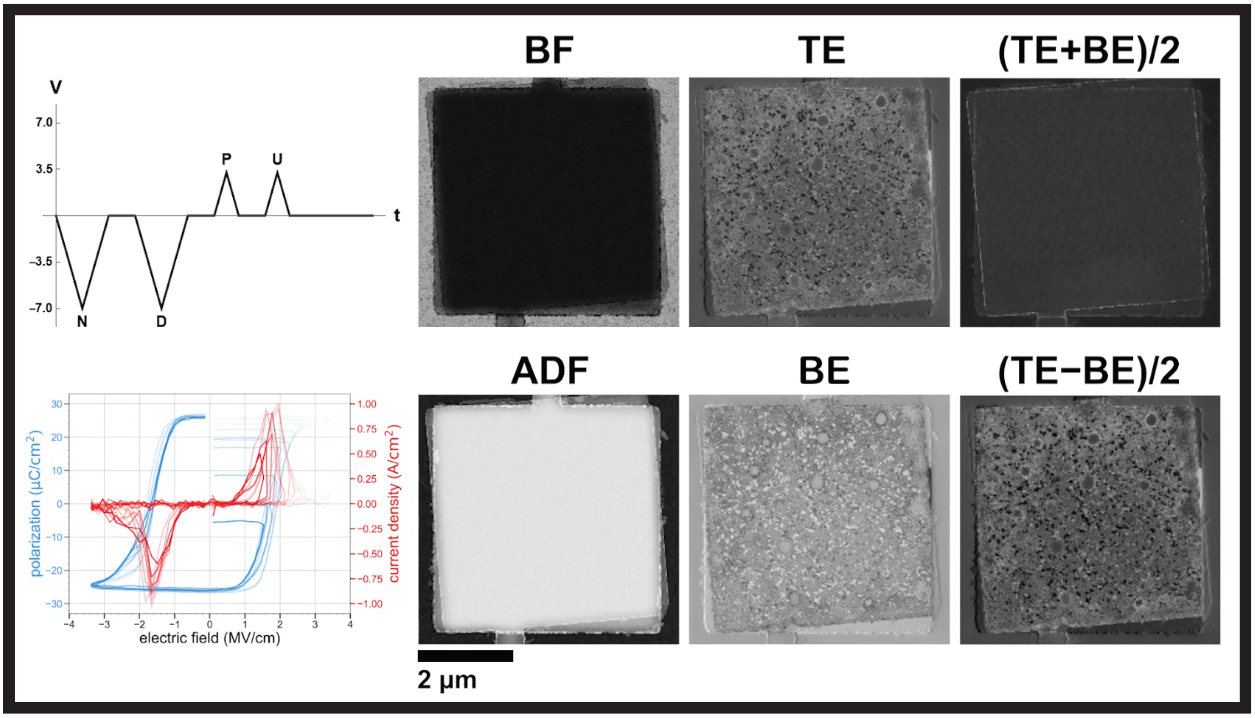

Movie M1: **Device-scale imaging of polarization switching.** One representative frame from the middle of the movie is shown above. The plot in the upper left corner shows a summary of the pulses that nominally set the polarization state. Here the summarized pulse sequence is PUNDp3.5, *i.e.,* two 7-V PUND waveforms followed by two-and-a-half 3.5-V PUND waveforms (Fig. S1). To the right are shown the STEM images that are acquired immediately after the summarized pulse sequence: bright field (BF), annular dark field (ADF), EBIC collected from the top electrode (TE), and EBIC collected from the bottom electrode (BE). The images are corrected for drift (both spatial and transimpedance amplifier offset) but otherwise unprocessed. Also shown are even and odd linear combinations of the EBIC images. Most of the EBIC contrast appears in the odd linear combination (TE−BE)/2, indicating that most of the EBIC is produced by electron-hole pair separation. Note that the electron-hole pair separation EBIC flows uniformly from one transimpedance amplifier through the capacitor to the other transimpedance amplifier.

The lower left corner shows a subset of the transport data collected immediately before the images shown. The current density $J(E)$ and its integral — the polarization $P(E)$ — shown are taken from the pulses in box "C" in Fig. S1a, which contains the second half of the second init pulse sequence and the first half of the first var sequence. In other words, the current and polarization shown reflect the voltage pulses shown in the summary plots (upper left corner) directly above the $J(E)$ and $P(E)$ plots.

The PUND data shows obvious imprint effects. As the var voltages decrease, the PUNDp (NDPUn) current peaks both shift left (right) relative to $E = 0$. Imprinting, which happens as a domain soaks for a while at a given polarization polarity, makes the first polarization reversal harder and the second reversal (back to the imprinted state) easier. The resultant splitting of the current peak is even more striking in the first init pulse sequence (not shown).

The movie shows the full, 40-point dataset twice. First, the data is shown in the order that it was acquired. Because the data was acquired by alternating between PUNDp and NDPUn, the de-interleaved PUNDp and NDPUn datasets are then shown separately. As the variable voltage magnitude varies in 19 steps from 7 V to 0 V, the PUNDp (NDPUn) series shows the ferroelectric polarization varying from maximal $P_\uparrow$ ($P_\downarrow$) to maximal $P_\downarrow$ ($P_\uparrow$).

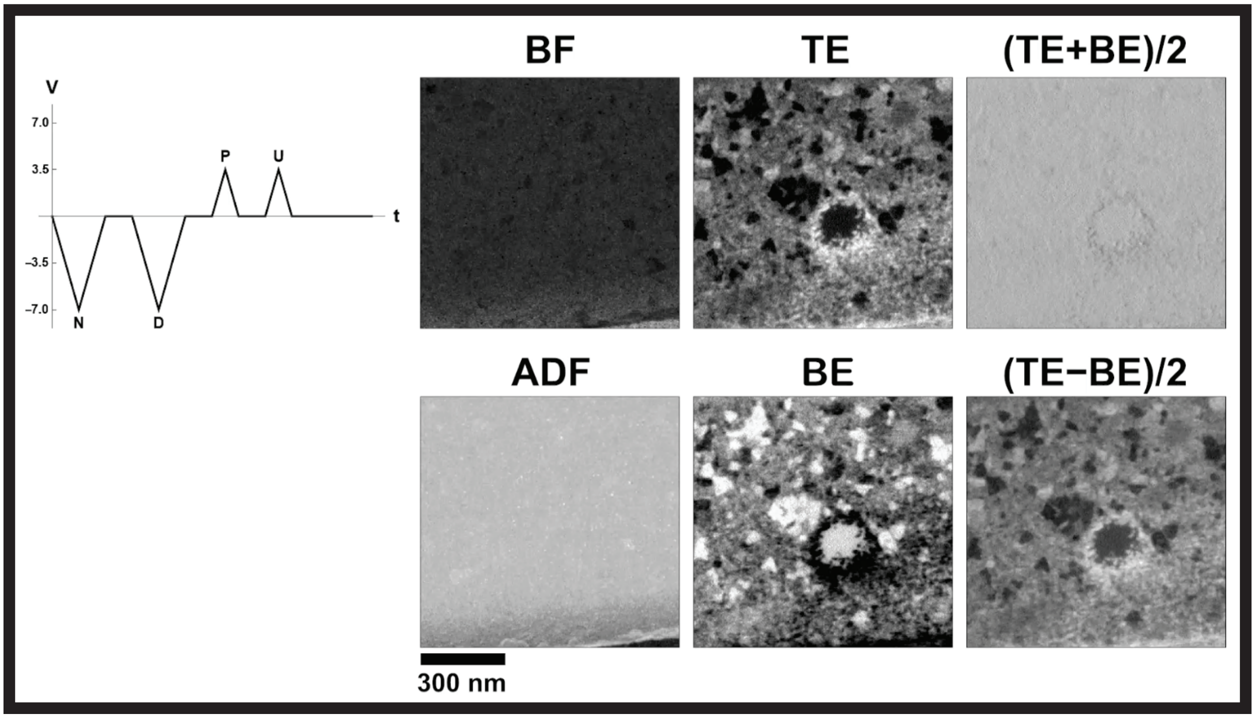

Movie M2: **Domain-scale imaging of polarization switching.** One representative frame from the middle of the movie is shown above. The plot in the upper left corner shows a summary of the pulses that nominally set the polarization state. Here the summarized pulse sequence is PUNDp3.5, *i.e.,* two 7-V PUND waveforms followed by two-and-a-half 3.5-V PUND waveforms. To the right are shown the STEM images that are acquired immediately after the summarized pulse sequence: bright field (BF), annular dark field (ADF), EBIC collected from the top electrode (TE), and EBIC collected from the bottom electrode (BE). The images are corrected for drift (both spatial and transimpedance amplifier offset) but otherwise unprocessed. Also shown are even and odd linear combinations of the EBIC images. See the caption of Movie M1 for further discussion. As in Movie M1, transport data (which reflects the whole device) are acquired immediately before each of these image sets, but they are not shown here because the area imaged is only 4% of the complete device and therefore non-representative.

The movie shows the full, 34-point dataset twice. First, the data are shown in the order that they were acquired. Because the data were acquired by alternating between PUNDp and NDPUn, the de-interleaved PUNDp and NDPUn datasets are then shown separately. As the variable voltage magnitude varies in 16 steps from 7 V to 0 V, the PUNDp (NDPUn) series shows the ferroelectric polarization varying from maximal $P_\uparrow$ ($P_\downarrow$) to maximal $P_\downarrow$ ($P_\uparrow$).

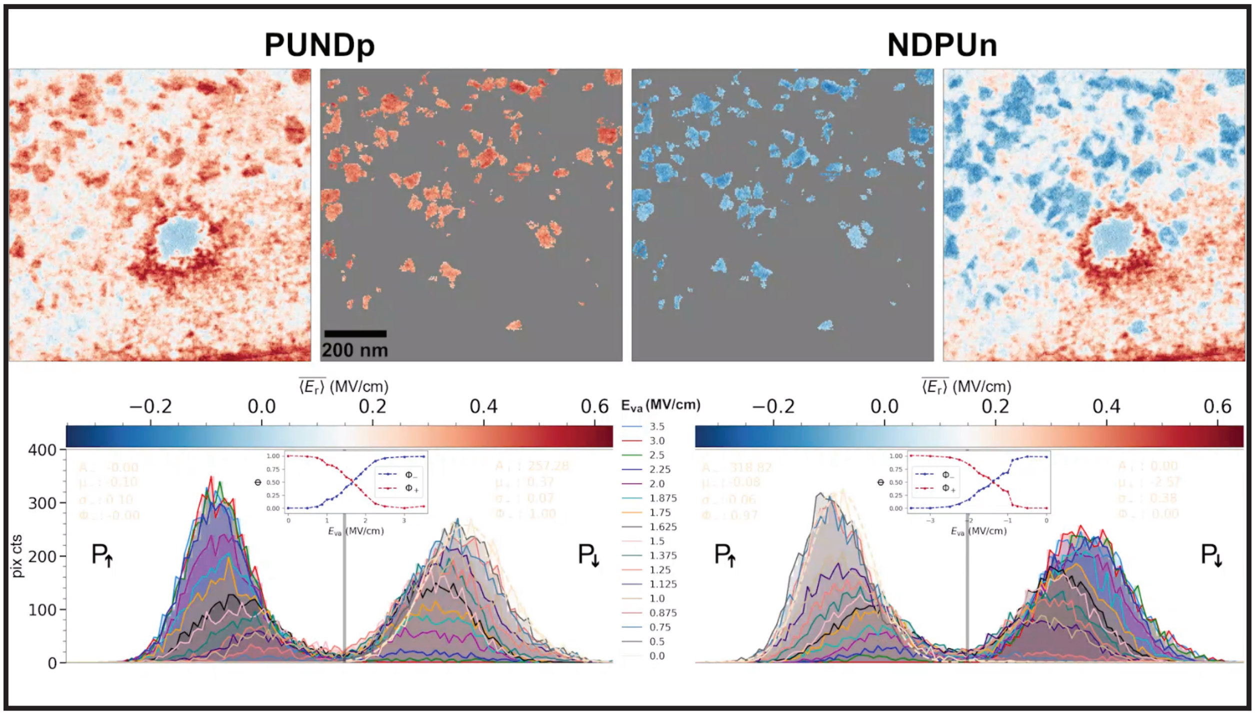

Movie M3: **Remanent field $\langle E_{\mathbf{r}} \rangle$ evolution with set voltage V.** One representative frame from the end of the movie is shown above. This movie is generated using the STEM EBIC imaging data shown in Movie M2. The outer maps (top row) show the exact same (TE-BE)/2 data, only with a blue-white-red intensity scale that is calibrated in MV/cm (Fig. S3). In other types of electron microscopy, currents of only one sign (*e.g.,* electrons) are detected, but in EBIC imaging both positive and negative currents (electrons and holes) are detected. This difference makes three-color intensity scales, where positive, negative, and zero can be clearly distinguished, more appropriate than the usual grayscale. Here the three-color scale makes it easier to distinguish the two polarization states from each other, and from the unpolarized/non-ferroelectric condition. The background has not been subtracted, so an overall bias toward $P_{\downarrow}$ is evident. The switching portion of the remanent electric field $\langle E_{\mathrm{r}} \rangle$ is, of course, opposite P.

The inner maps show the regions that switch once and only once in both the PUNDp and NDPUn datasets — everything else is masked (Fig. S13). As the movie proceeds from large set voltages to small set voltages, the domain populations switch from entirely $P_{\uparrow}$ ($P_{\downarrow}$) for PUNDp (NDPUn) to entirely $P_{\downarrow}$ ($P_{\uparrow}$), as seen in histograms of the inner maps (bottom row). Fits to the Gaussian function $A\exp[(\langle E_{\mathrm{r}} \rangle - \mu)^2/2\sigma^2]$ reveal this trend quantitatively. The histogram insets plot the normalized populations $\Phi = (\sigma A)/(\sigma_{\max} A_{\max})$. Plotting the means μ (Fig. S17) shows quantitatively what is evident by eye here: regions with more negative $\langle E_{\mathrm{rb}} \rangle$ require more positive E_{va} to switch to $P_{\uparrow}$ and less negative E_{va} to switch to $P_{\downarrow}$. Further analysis (Movie M4) shows this same effect at the individual domain level (Fig. S18).

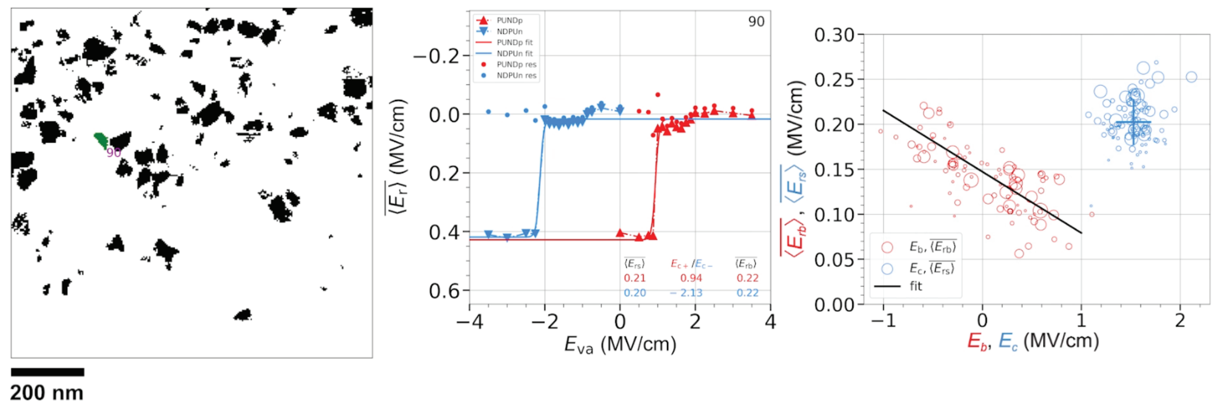

Movie M4: **Domain-by-domain switching behavior. (left)** One representative frame from the movie is shown above. A mask shows the regions that switch once and only once (the white regions in Fig. S13) in both the PUNDp and NDPUn datasets (Movies M2, M3). Contiguous domains labeled with numbers 0–97 are highlighted as their EBICs are fit. **(middle)** The remanent electric field $\overline{\langle E_r \rangle}$, averaged over each domain, is fit as a function of the var applied field E_{va}. The fit function is $\overline{\langle E_r \rangle} = \overline{\langle E_{rs} \rangle} \tanh\left[\frac{E_{va} - E_{c\pm}}{0.1 \text{ MV/cm}}\right] + \overline{\langle E_{rb} \rangle}$ with fit parameters $\overline{\langle E_{rs} \rangle}$, $E_{c\pm}$, and $\overline{\langle E_{rb} \rangle}$. The fit parameter $E_{c\pm}$ is E_{c+} or E_{c-}, depending on whether the PUNDp or the NDPUn data, respectively, are being fit. The $\overline{\langle E_r \rangle}$-axis is inverted to make the similarity with a one-domain $P(E)$ curve obvious (P and $\langle E_{rs} \rangle$ have opposite sign). The label of the domain being fit is shown in the upper right corner, and the parameters returned by the fits are shown in the lower right corner. Overall, the tanh function fits are very successful, showing that every domain (and indeed every switching pixel — see Movie M5 and Fig. S20) makes an abrupt, single-data-point transition between the polarization states. For comparison see Fig. 3 of Ref. 29. **(right)** As the domains are fit their $[E_c, \overline{\langle E_{rs} \rangle}]$ (blue) and $[E_b, \overline{\langle E_{rb} \rangle}]$ (red) are plotted , where the unbiased coercive field E_c and the coercive field bias E_b are defined by $E_{c\pm} = E_b \pm E_c$. The domains are sorted according to their $\overline{\langle E_{rb} \rangle}$. See Fig. S18 for further discussion.

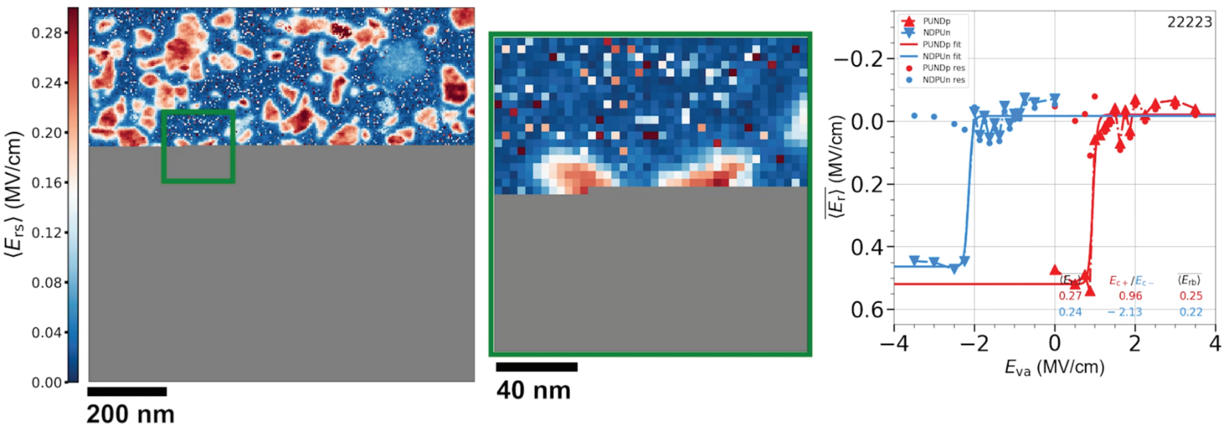

Movie M5: **Pixel-by-pixel switching behavior.** This movie is analogous to Movie M4 and created from the same dataset (Movie M2), but in this case we are not confining the fitting to the domains that switch once and only once, and we are not averaging $\langle E_{\rm r}\rangle$ over each domain before fitting. The fit function is $\langle E_{\rm r}\rangle = \langle E_{\rm rs}\rangle \tanh\left[\frac{E_{\rm va}-E_{c\pm}}{0.1\ {\rm MV/cm}}\right] + \langle E_{\rm rb}\rangle$ with fit parameters $\langle E_{\rm rs}\rangle$, $E_{c\pm}$, and $\langle E_{\rm rb}\rangle$. Here we only map $\langle E_{\rm rs}\rangle$, but maps of all of the four fit parameters are given in Fig. S20 Even at eight frames per second, showing the fits for each pixel would take more than two hours, so we display a random set of pixels plus all of the pixels in an example domain (#90), chosen at random, from Movie M4.

SI.5 Supplementary Figures

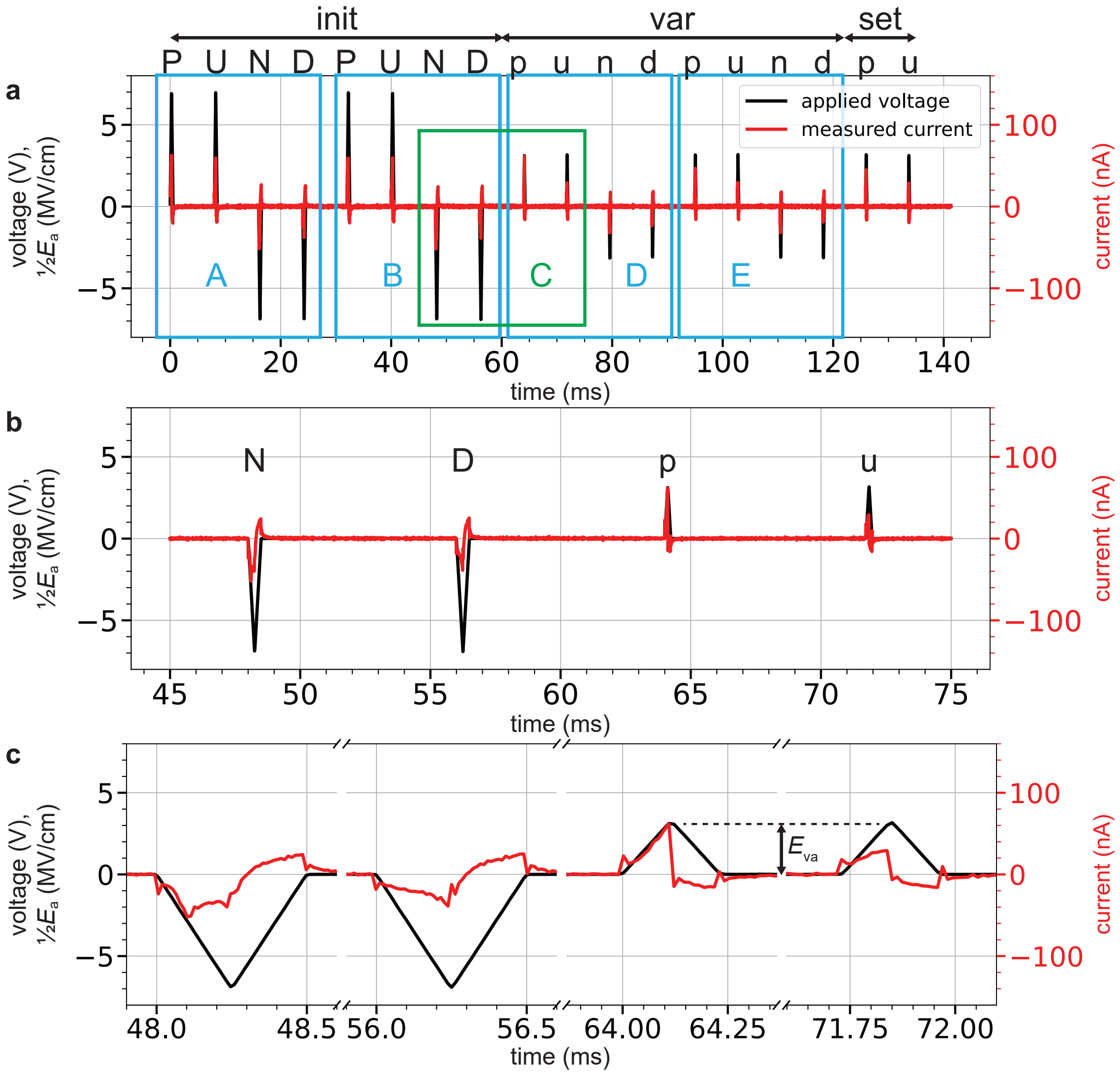

Figure S1: **PUND sequence.** **(a)** A full PUNDp3.25 sequence, showing two 7 V PUND waveforms followed by two-and-a-half 3.25 V PUND waveforms. The different boxed regions A–E construct the different $P(E)$ loops in Fig. S2 with their corresponding labels. **(b)** A zoomed view of the "C" region boxed in (a). **(c)** The same time period shown in (b), but without the 7.5 ms delays between triangular pulses.

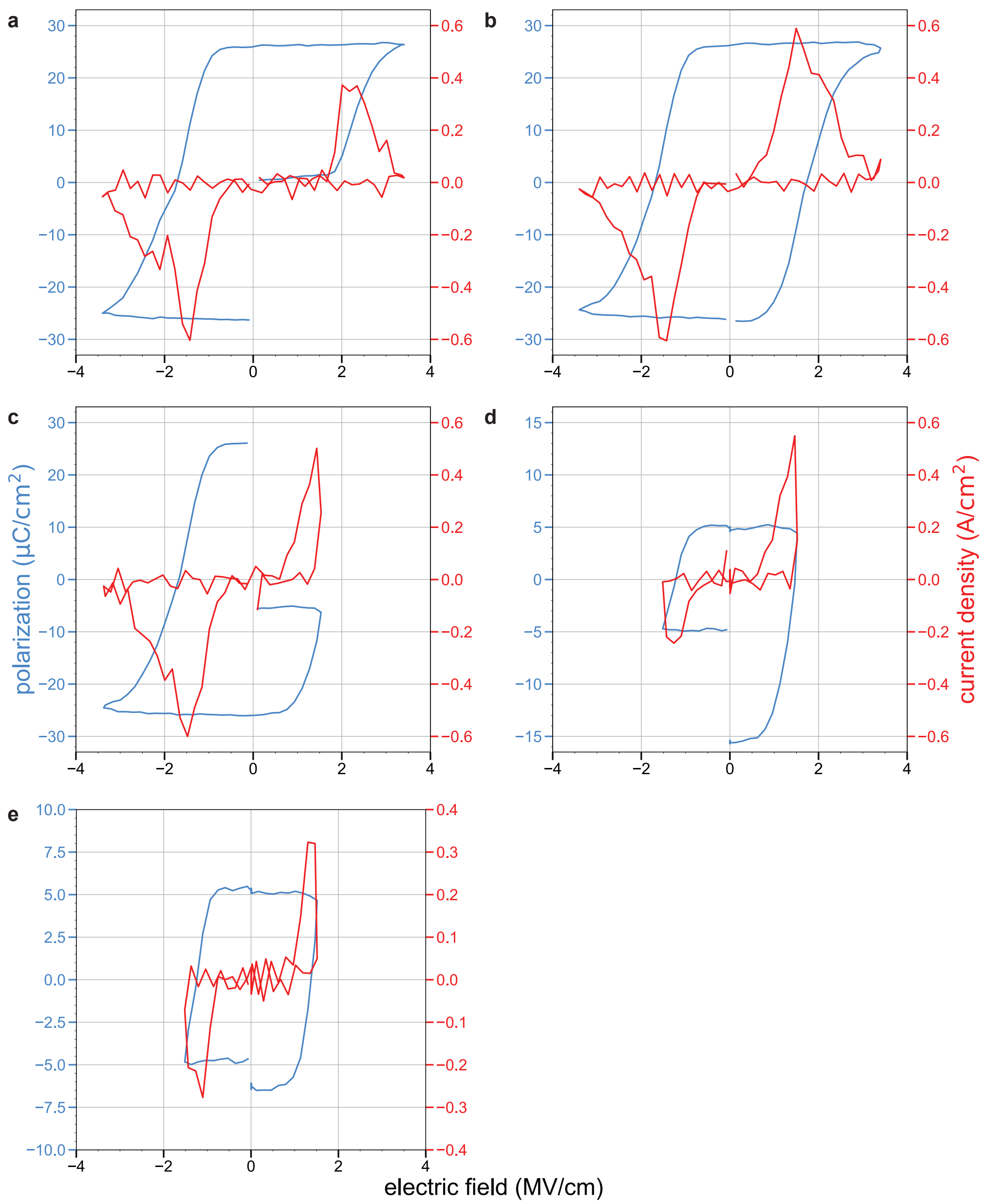

Figure S2: **PUND-derived $P(E)$ loops.** These hysteresis curves **(a–e)** are generated from the corresponding boxed sections A–E of the PUNDp3.25 sequence in Fig. S1a. Each polarization curve $P(E)$ includes an unknown constant of integration. We center the $P(E)$ curves by taking the difference between the halfway $E = 0$ point and either the initial *or* the final integrated polarization to be twice the initial polarization. The plots in Fig. 2e are analogous to those of part (e) here, while the blue triangles in Fig. 2f are generated by averaging the last 10 points (11.2 mV) before $E = 0$ at the *end* of the blue curve in part (c) here.

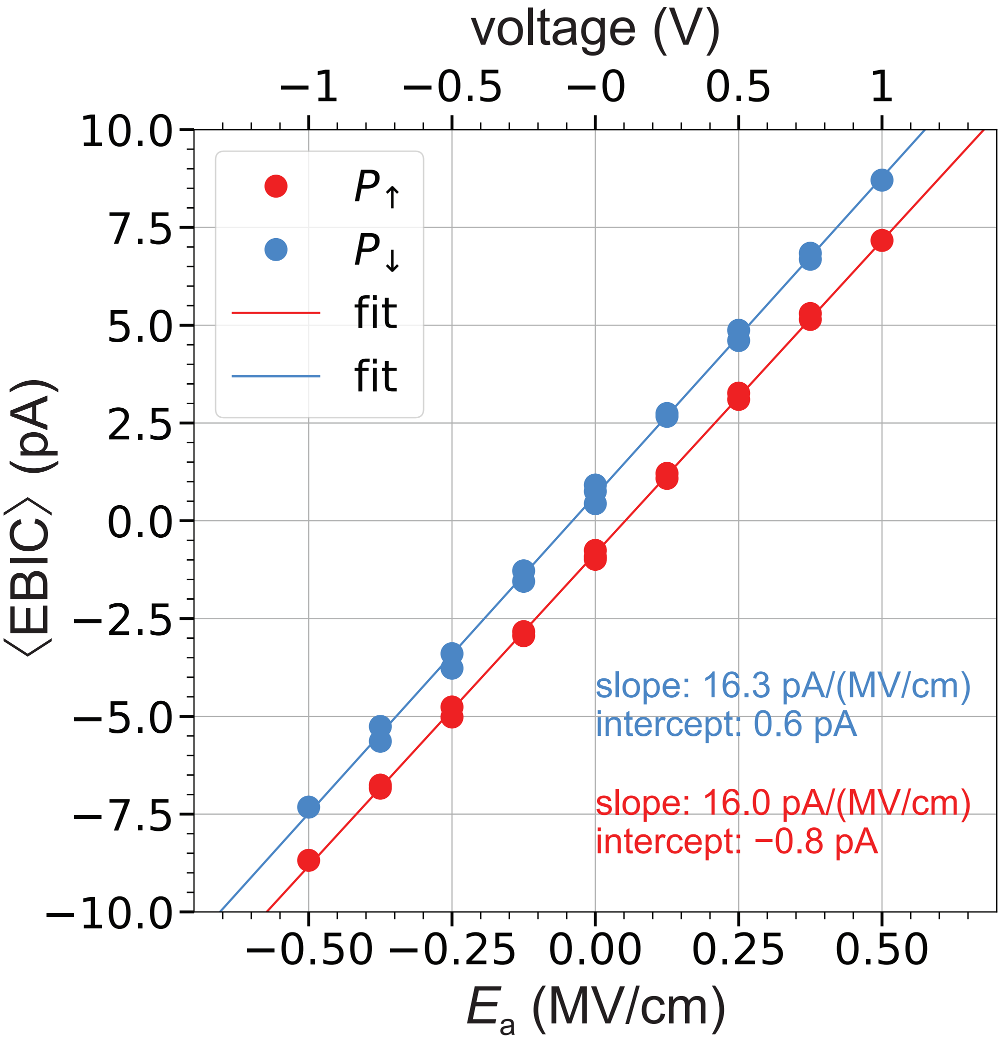

Figure S3: **EBIC vs. E-field calibration.** Using a battery, we adjust the "common" potential of the BE TIA such that it is at a voltage V relative to the common of the TE TIA. Thus the BE is held at a virtual potential V relative to the TE, which is held at virtual ground. We then acquire EBIC images with the field of view used for Figs. 2a–d and plot the EBIC $(I_{\text{top}} - I_{\text{bot}})/2$ averaged over the capacitor's active area (Fig. S7c). The EBIC is also linear in the beam current (150 pA for this data), so we take the local electric field $\langle E \rangle$ to be $\langle E \rangle = 9.3[\text{MV/cm}]\frac{I_{\text{top}} - I_{\text{bot}}}{2\, I_{\text{beam}}}$.

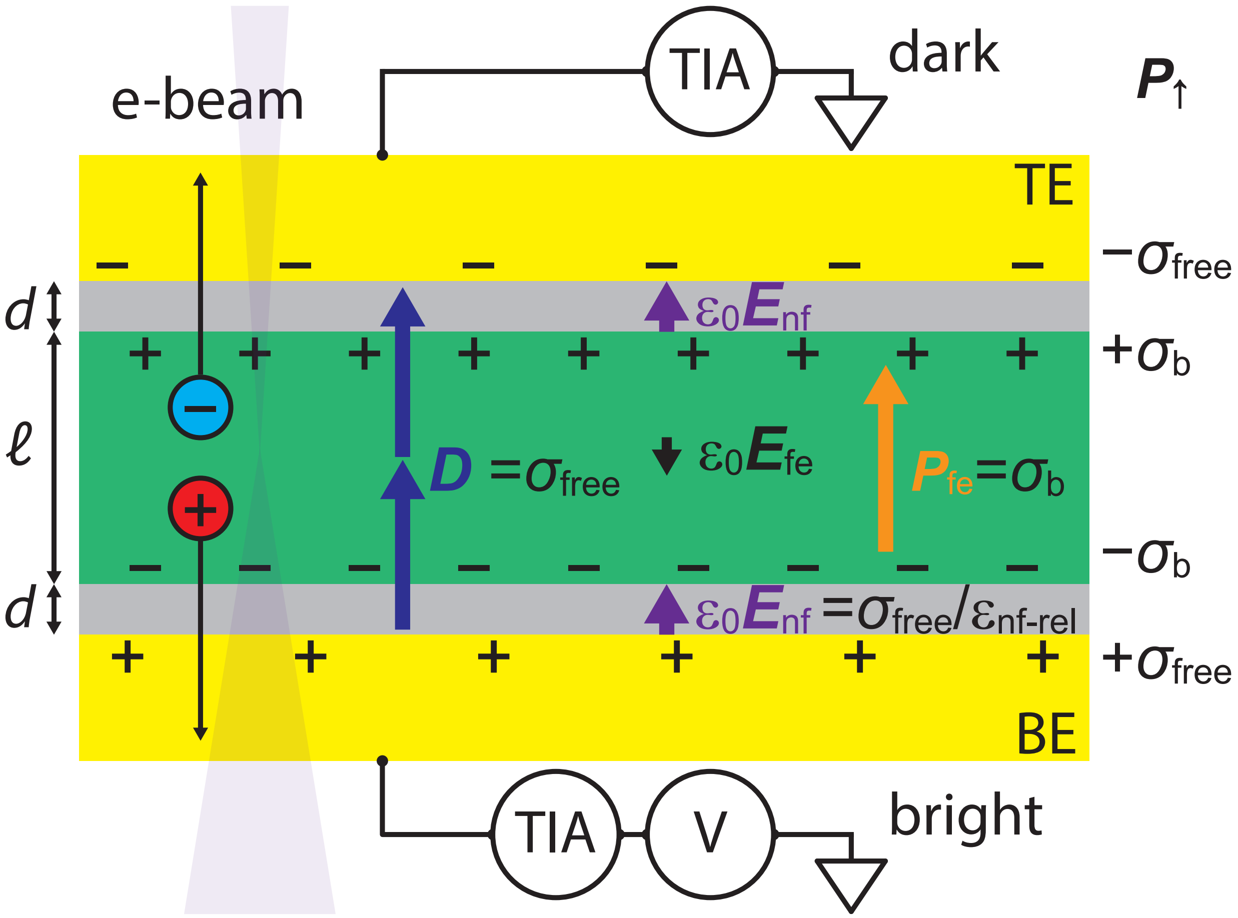

Figure S4: **Field geometry and contrast model.** Transimpedance amplifiers (TIAs) are connected to the top electrode (TE) and bottom electrode (BE). The input of the BE TIA can be displaced by a voltage V from the potential of the input of the TE TIA, which is held at virtual ground. The charge distribution shown here shows a ferroelectric (green) of thickness ℓ in the polarization $P_\uparrow$ state with $V = 0$. The ferroelectric's polarization is equal to the bound surface charge density ($P_{\rm fe} = \sigma_{\rm b}$). This charge is partially screened by a free surface charge density $\sigma_{\rm free}$ in the electrodes (yellow). The screening is incomplete (Eq. S8) because of non-ferroelectric layers of thickness d at the electrode-ferroelectric interfaces. Because there is no free charge in the ferroelectric, the displacement field $D = \sigma_{\rm free}$ is continuous from the top of the BE to the bottom of the TE. See sections SI.1–SI.3 for further discussion.

The electron beam generates electron (blue) - hole (red) pairs in the ferroelectric. For $P_\uparrow$ the net remanent electric field $E_{\rm fe}$ is negative, which drives the electrons toward the TE and the holes toward the BE. The resultant EBIC can be thought of as a continuous (conventional) current from the TE to the BE. Positive current is displayed as bright contrast, so this EBIC $= (I_{\rm top} - I_{\rm bot})/2$ appears as dark contrast in an image (*e.g.*, Fig. 2a).

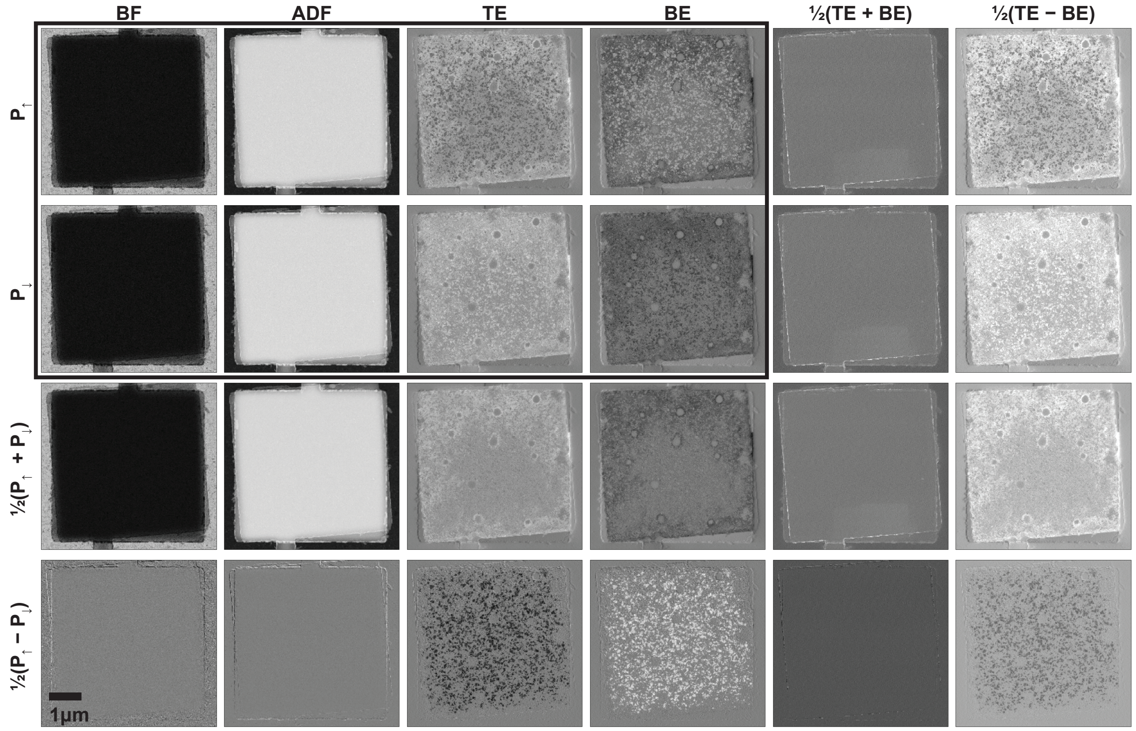

Figure S5: **Whole-device imaging of $P_{\updownarrow}$ states with BF, ADF, and STEM EBIC.** The first two rows of the first four columns (boxed in black) show raw data. The first (second) row shows simultaneously acquired images of the maximum $P_{\uparrow}$ ($P_{\downarrow}$) state after a +7 V (−7 V) set pulse has been applied. The standard bright-field (BF) and annular dark-field (ADF) STEM images show no appreciable changes when the capacitor is switched between the $P_{\uparrow}$ and the $P_{\downarrow}$ states. The STEM EBIC images acquired from TIAs connected to the top electrode (TE) and bottom electrode (BE), on the other hand, show changes that are easily visible in the raw data. The fifth (sixth) column separates out the EBIC contrast that is common (opposite) between the two electrodes. Most of the EBIC contrast appears in the (TE-BE)/2 column and arises from electron-hole pair separation generated by electric fields inside the sample. This column shows, without auto-contrasting, the same images that are shown in Fig. 2. (The third and fourth columns, second row, show the same images that are shown in Fig. 1.) Contrast in the (TE+BE)/2 column is primarily generated by SEEBIC. Evidence of the higher-magnification imaging (Fig. S6), probably an effect created by charging by the beam (it eventually goes away without any baking or cleaning), is evident in the first three rows of the (TE+BE)/2 column. The third (fourth) row separates out the contrast that is common (opposite) between the two polarization states, $P_{\uparrow}$ and $P_{\downarrow}$. The switchable ferroelectric domains appear with dark contrast in the rightmost image of the last row.

In the EBIC images, spot-like $\sim$ 100-nm-radius features generating little signal are scattered about the field of view. In the standard STEM images these features are completely invisible. These spots are likely regions that do not make good electrical contact to the TE, because they give an EBIC response to an applied voltage V like that of the rest of the capacitor (Fig. S3), but with a smaller slope. The bad connections seem to be at the TE because the EBIC contrast in these regions at $V = 0$ is closer to that of the BE alone than to that of the TE alone. These spots illustrate how STEM EBIC imaging can reveal and diagnose defects impacting device function that standard STEM does not even detect.

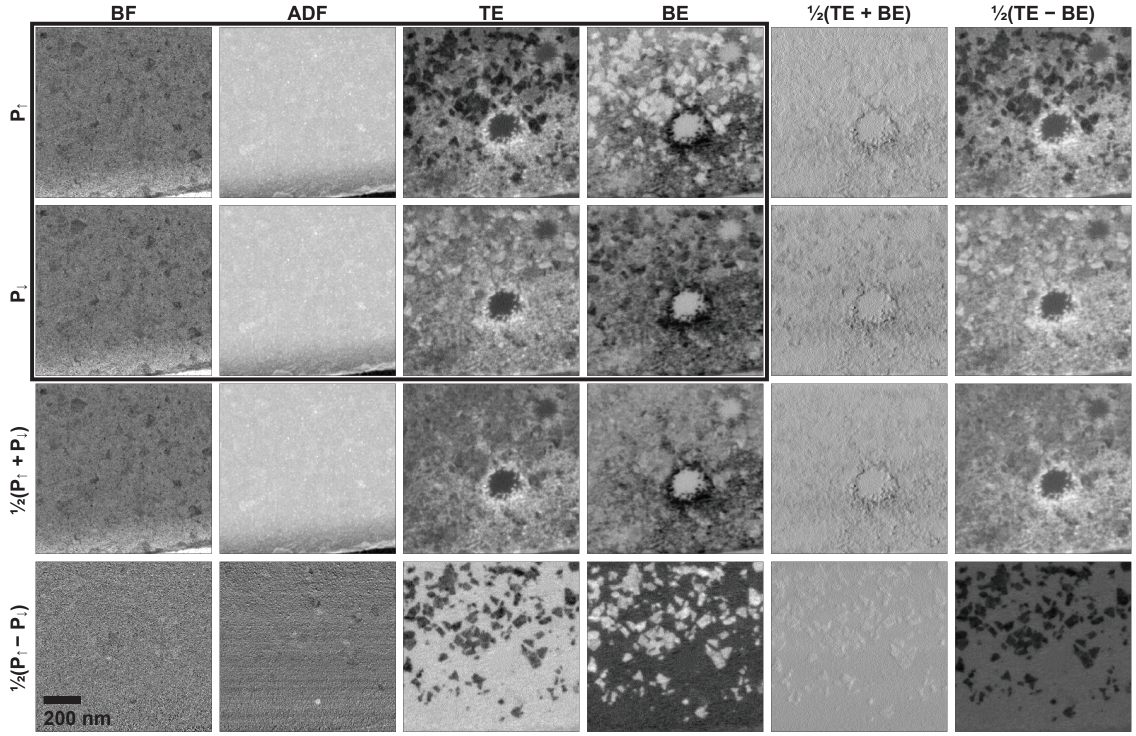

Figure S6: **Domain-scale imaging of $P_{\updownarrow}$ states with BF, ADF, and STEM EBIC.** The first two rows of the first four columns (boxed in black) show raw data. The first (second) row shows simultaneously acquired images of the maximum $P_{\uparrow}$ ($P_{\downarrow}$) state after a +7 V (−7 V) set pulse has been applied. The standard bright-field (BF) and annular dark-field (ADF) STEM images show no appreciable changes when the capacitor is switched between the $P_{\uparrow}$ and the $P_{\downarrow}$ states. The STEM EBIC images acquired from TIAs connected to the top electrode (TE) and bottom electrode (BE), on the other hand, show changes that are easily visible in the raw data. The fifth (sixth) column separates out the EBIC contrast that is common (opposite) between the two electrodes. Most of the EBIC contrast appears in the (TE-BE)/2 column and arises from electron-hole pair separation generated by electric fields inside the sample. We expect the contrast in the (TE+BE)/2 column to be primarily generated by SEEBIC. This particular dataset is acquired with external EBIC TIAs (Methods), and their gains are not perfectly matched, so some E-field related contrast leaks into the (TE+BE)/2 channel. In comparison, the data of Fig. S5, which is acquired with internal amplifiers, shows no E-field related contrast in the (TE+BE)/2 channel. The third (fourth) row separates out the contrast that is common (opposite) between the two polarization states, $P_{\uparrow}$ and $P_{\downarrow}$. In the EBIC images, one of the 100-nm-radius, zero-EBIC spots (Fig. S5) is clearly visible just below the center of the field of view. Even at this higher magnification, this feature is still completely invisible to the standard STEM imaging detectors. The switchable ferroelectric domains appear with dark contrast in the rightmost image of the last row.

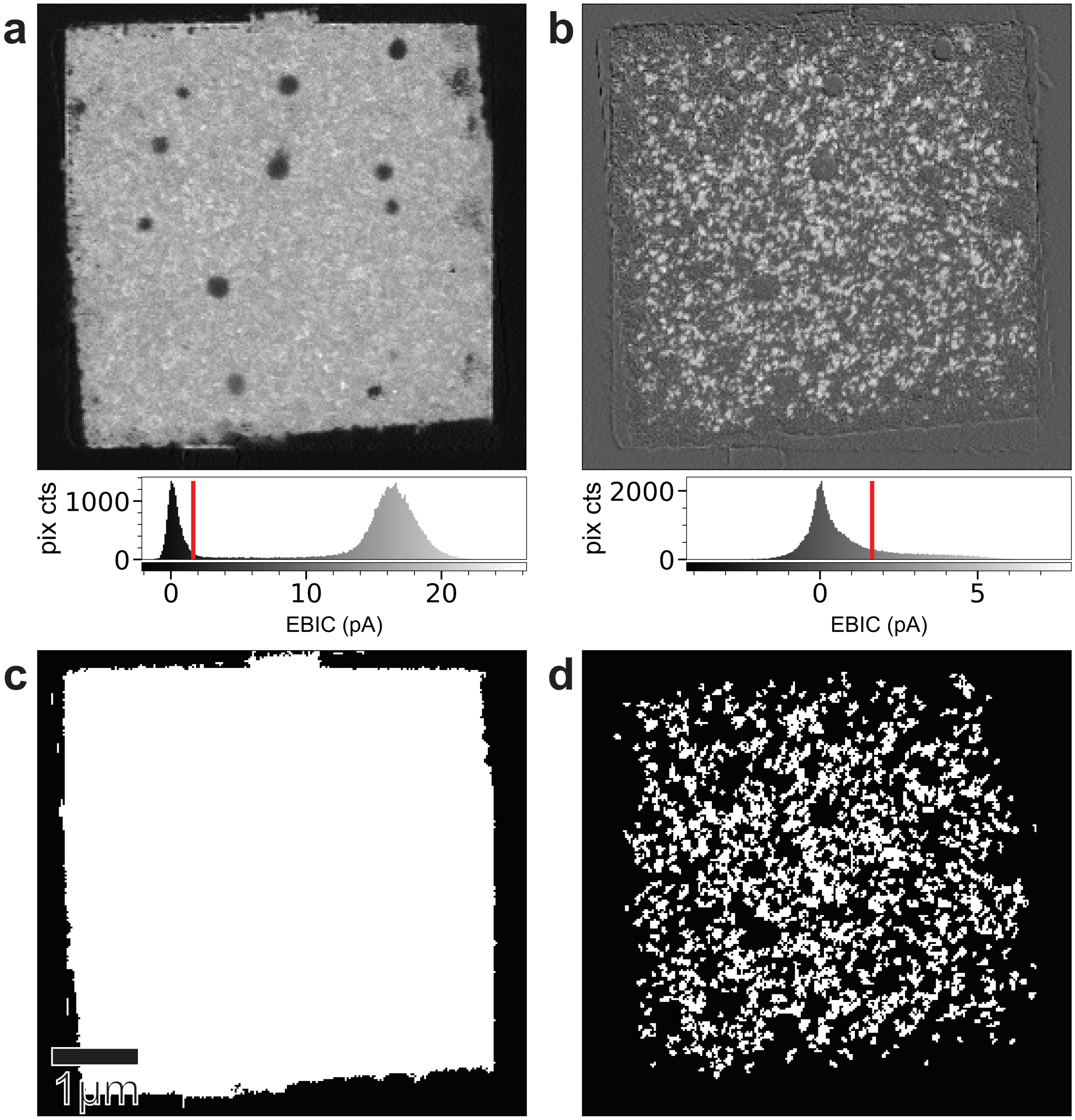

Figure S7: **Device-scale capacitor and switching masks.** **(a)** A linear combination of EBIC images acquired while the capacitor is under ±1 V for both the $P_\uparrow$ and the $P_\downarrow$ states. Representing an image $\mathtt{Im}$ as $\mathtt{Im}(V, P)$, here we show $[\mathtt{Im}(+1,\uparrow) + \mathtt{Im}(+1,\downarrow) - \mathtt{Im}(-1,\uparrow) - \mathtt{Im}(-1,\downarrow)]/4$. These four images are like those of the calibration dataset (Fig. S3), but 256 × 256 pixels instead of 128 × 128 pixels. **(b)** The linear combination [NDPUn7 − PUNDp7 + PUNDp0 − NDPUn0]/4 of EBIC images from the Fig. 2 dataset. This combination highlights the switchable domains. The capacitor mask **(c)** and switching mask **(d)** are obtained by thresholding (a) and (b), respectively, at the currents indicated by red vertical bars on the histograms. The threshold that defines the capacitor mask (c) is chosen to capture the small-EBIC spots visible in (a), because these spots (1.5% of the capacitor area) contribute to the device capacitance (Figs. S5–S6).

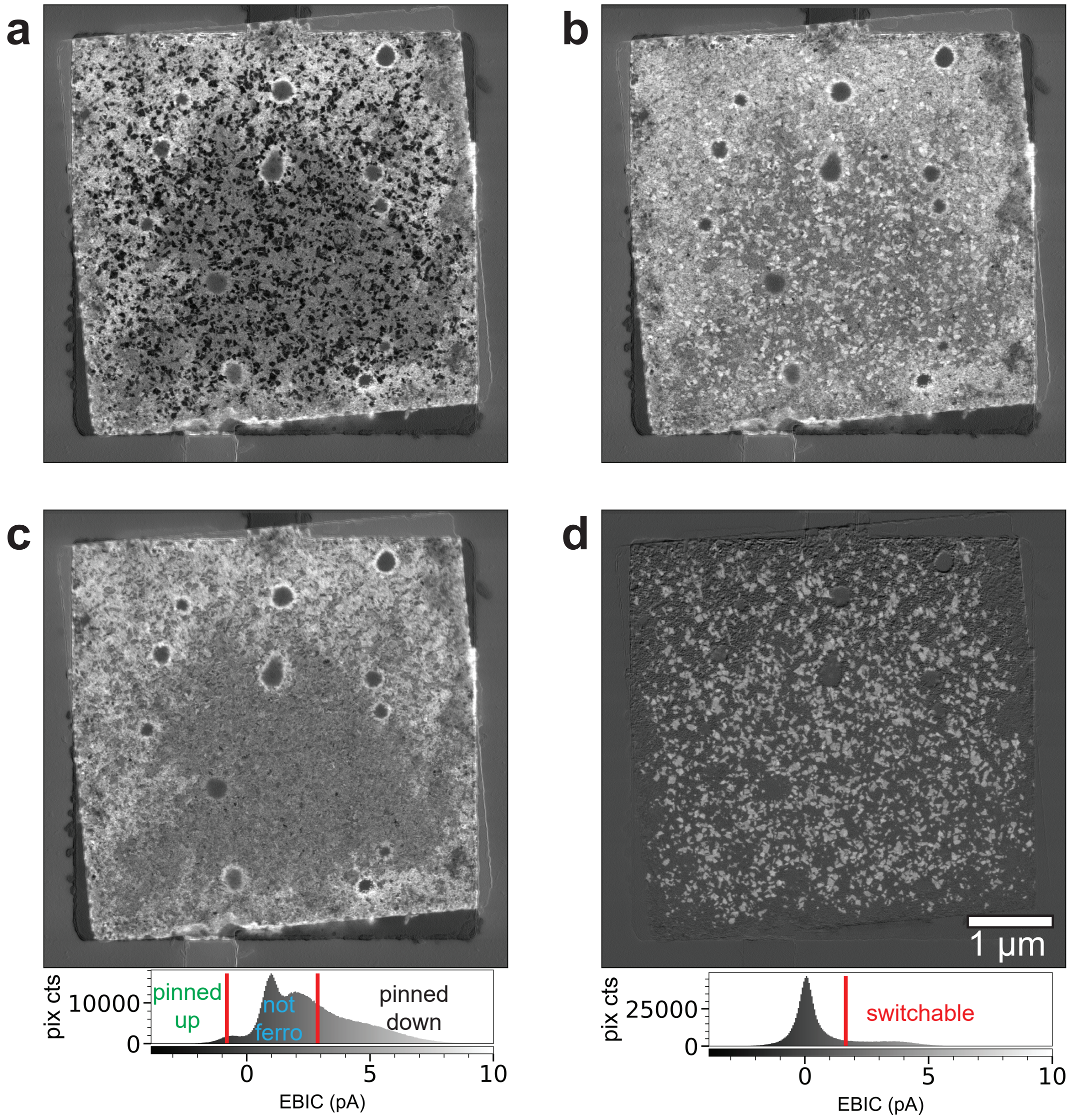

Figure S8: **Device-scale high-resolution EBIC images.** These 1024 pixel × 1024 pixel EBIC images are analogous to the images shown in Fig. 2a–d, but with 16× the number of pixels. **(a)** shows the capacitor after PUNDp7, *i.e.,* in the maximal $P_{\uparrow}$ state. **(b)** shows the capacitor after NDPUn7, *i.e.,* in the maximal $P_{\downarrow}$ state. **(c)** and **(d)** show the sum $[(P_{\downarrow} + P_{\uparrow})/2]$ and difference $[(P_{\downarrow} - P_{\uparrow})/2]$ images, respectively. Red vertical lines on the histograms indicate where the thresholds are set to define switching, pinned up, pinned down, and non-switching in Fig. S11. Parts (a–d) are *not* auto-contrasted, but are shown using the same contrast limits (indicated by the intensity scale bar along the histograms' x-axes). The tallest peak in the (c) histogram is due to signal from the HZO outside the capacitor. Part (c) here is, neglecting small SEEBIC contributions, effectively a map of the background remanent fields $\langle E_{\mathrm{rb}} \rangle$. Similarly, part (d) here is a map of the switching remanent fields $\langle E_{\mathrm{rs}} \rangle$.

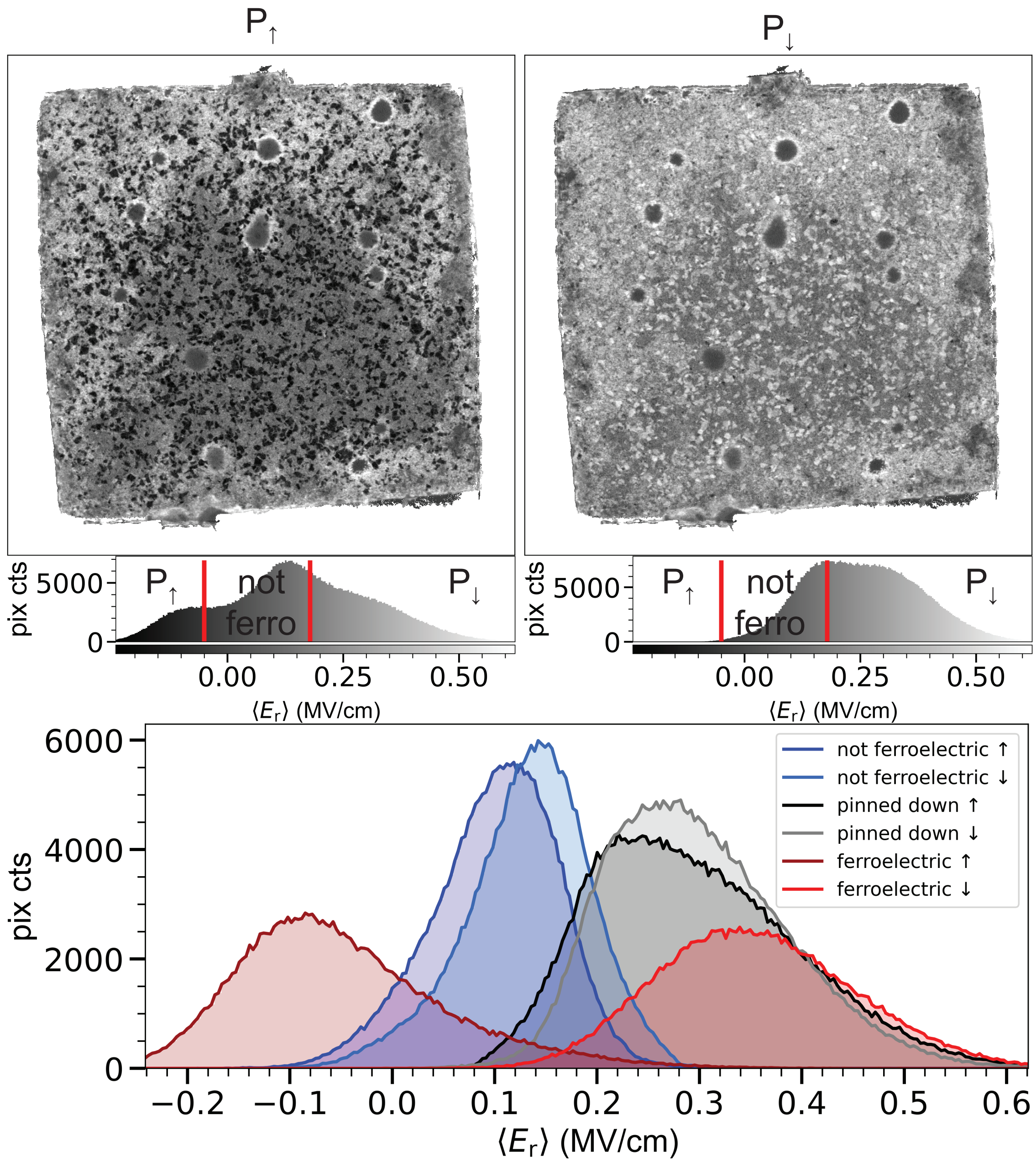

Figure S9: **Device-scale high-resolution $\langle E_\mathbf{r} \rangle$ distributions.** The $P_\updownarrow$ images shown here are the same as Fig. S8a–b, but with regions outside the electrode overlap masked. Histograms below each show the distribution of $\langle E_\mathrm{r} \rangle$ across the capacitor for the two polarization states, where the measured EBIC has been converted to E-field units using the calibration (Fig. S3). Vertical red bars on each histogram are identically located and demarcate (from left to right), polarized-up, non-ferroelectric, and polarized-down regions respectively. Polarized and switching regions are distinguished from polarized and pinned regions using a mask made from Fig. S8d using the threshold indicated there. The bottom histogram shows how successful this masking procedure is. The histograms for regions labeled as ferroelectric and switching (see Fig. S11) have little overlap between the $P_\uparrow$ and $P_\downarrow$ states, while the histograms for the non-ferroelectric and pinned regions shift only slightly.

The mean $\langle E_\mathrm{r} \rangle$ for the pinned down regions is smaller than the mean $\langle E_\mathrm{r} \rangle$ for the switchable regions in the $P_\downarrow$ state, implying that $\langle E_\mathrm{rb} \rangle$ is more negative in the pinned down regions (assuming that $\langle E_\mathrm{rs} \rangle$ is constant). We surmise that going to larger $|E_\mathrm{va}|$ than 3.5 MV/cm would continue the progression documented in Figs. S17–S19: these background fields $\langle E_\mathrm{rb} \rangle$ would be overcome and more of the pinned domains would become switchable. See Fig. S10 for further discussion.

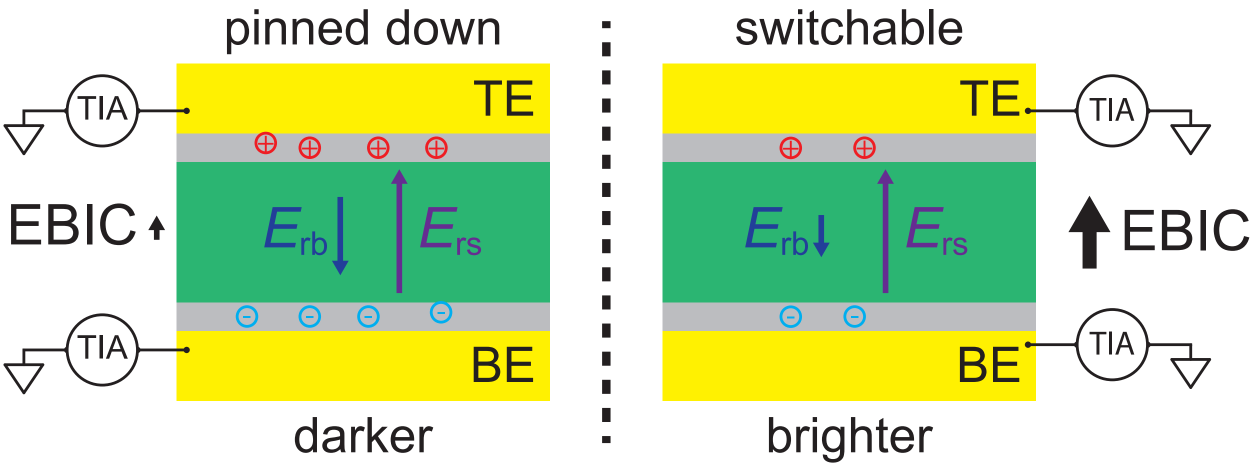

Figure S10: **Model of background field $\langle E_{\mathbf{rb}} \rangle$ effects on switching.** This cartoon shows graphically how the remanent fields $\langle E_{r\updownarrow} \rangle = \langle E_{rb} \rangle \mp \langle E_{rs} \rangle$ can be analyzed to understand and even predict which regions in a ferroelectric capacitor are switchable at a given voltage (see *e.g.*, Fig.S9). We take the switching remanent field $\langle E_{rs} \rangle$ to be a constant that is independent of position in the capacitor, an assumption well-supported by the narrowness of the measured E_c distribution (Figs. 4, S16). In this case, $P_\downarrow$ regions will have a smaller total (depolarizing) remanent field $\langle E_{r\downarrow} \rangle = \langle E_{rb} \rangle + \langle E_{rs} \rangle$ where the remanent background field $\langle E_{rb} \rangle$ is more negative, leading to a smaller EBIC **(left)**. Less negative $\langle E_{rb} \rangle$ leads to a more positive depolarizing field and easier switching to the $P_\uparrow$ state **(right)**. Thus we expect $P_\downarrow$ regions with smaller $\langle E_{r\downarrow} \rangle$ to be harder to switch to the $P_\uparrow$ state.

Our measurements match this expectation in multiple instances. The PUNDp data in Movie M3 shows both $\langle E_r \rangle$ histograms shifting to more positive values as the var voltage is decreased; the regions with the more negative $\langle E_r \rangle$ switch to $P_\uparrow$ only at the largest positive var pulses. Likewise, the NDPUn data in Movie M3 shows both $\langle E_r \rangle$ histograms shifting to more negative values as the var voltage is decreased; the regions with more positive $\langle E_r \rangle$ switch to $P_\downarrow$ only at the largest negative var pulses. These trends are shown quantitatively in Fig. S17.

“Pinned” domains represent the limiting case of this argument. Figure S9 shows a histogram with $\langle E_r \rangle$ distributions for ferroelectric and pinned domains separately. The mean $\langle E_r \rangle$ for ferroelectric $P_\downarrow$ regions is larger than that of the regions pinned in the $P_\downarrow$ state, implying that the pinned regions have more negative $\langle E_{rb} \rangle$. Presumably applying $V > 7$ V, the maximum previously applied, would unpin some of these domains.

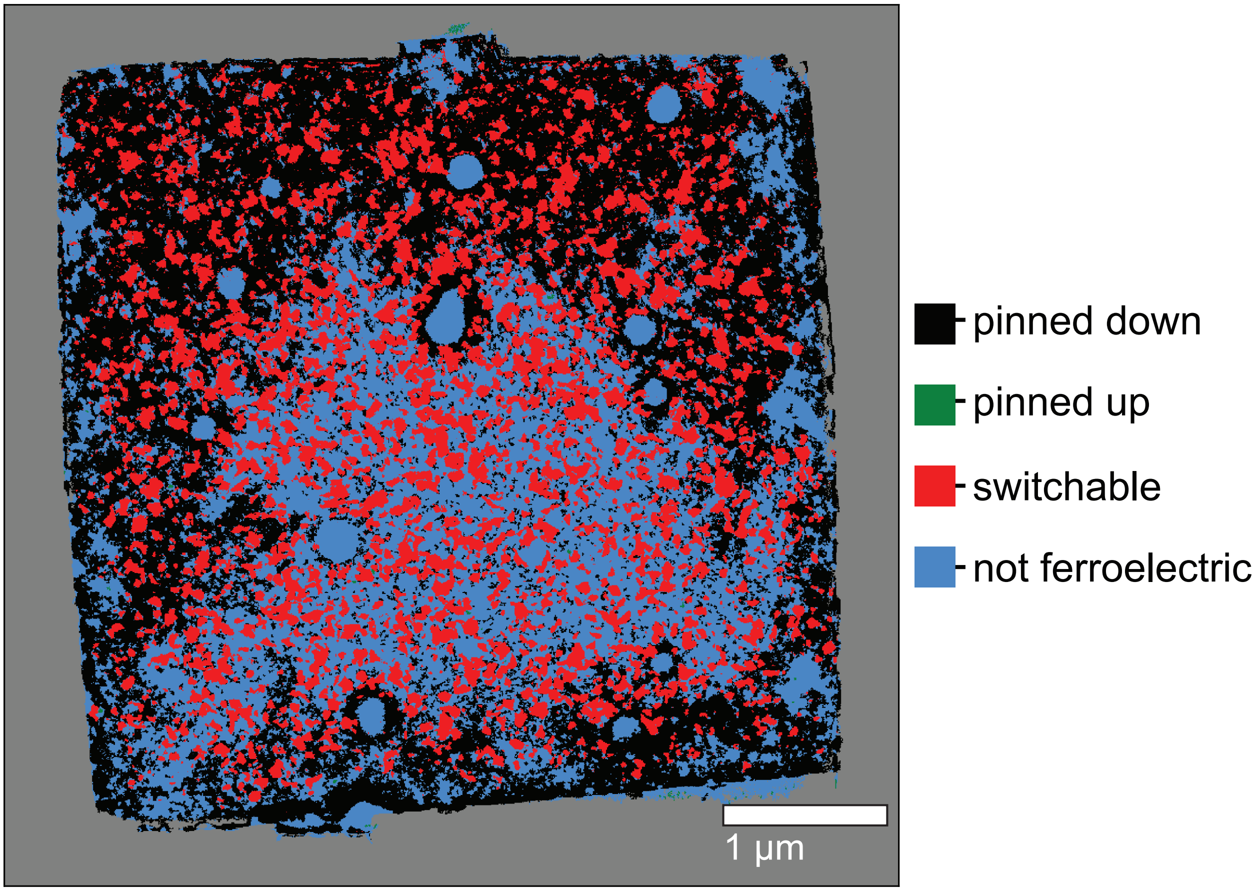

Figure S11: **Device-scale switching map.** The thresholds shown in Figs. S8–S9 are used to define switchable, pinned up, pinned down, and non-ferroelectric regions. Specifically, the switchable regions are defined by thresholding Fig. S8d at the point indicated by the red line in the accompanying histogram. The regions that are not switchable are partitioned into pinned up, not ferroelectric, and pinned down by thresholding Fig. S8c at the points indicated by the red lines in its accompanying histogram.

The switchable regions (red, 180759 pixels, 24.5% of the capacitor area) are dispersed relatively uniformly across the capacitor, with larger domains perhaps being less common near the edge of the capacitor. In contrast, pinned down (black, 264915 pixels, 35.9%) and non-ferroelectric (blue, 290939 pixels, 39.4%) regions show definite clustering. The amount of the capacitor area that is pinned up (green, 951 pixels, 0.1%) is so small as to be indistinguishable from noise.

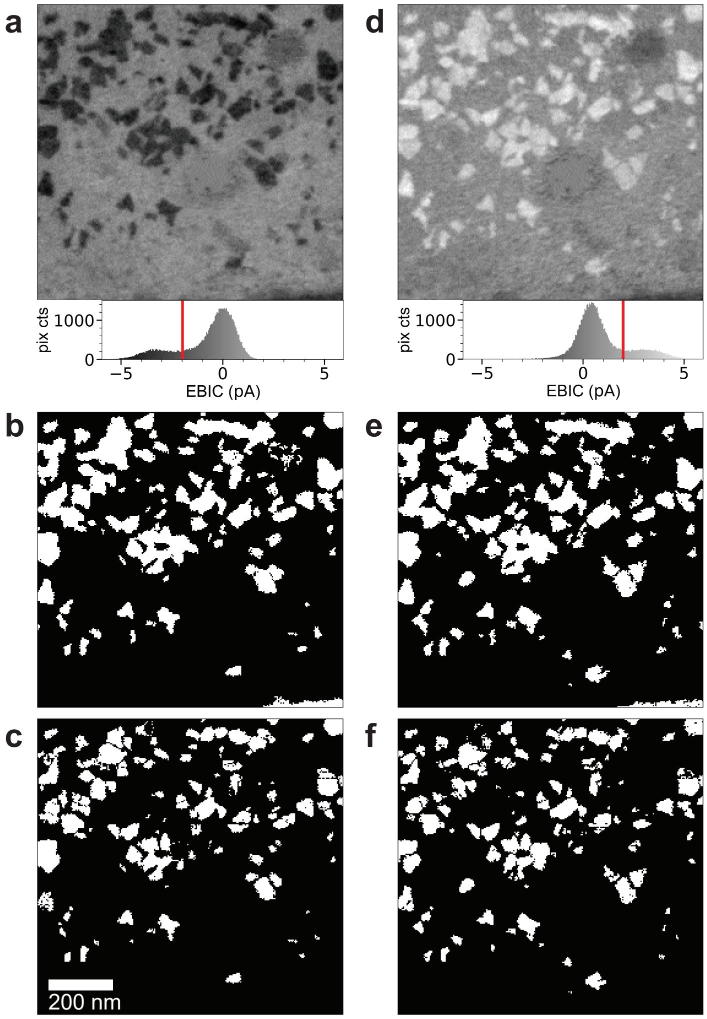

Figure S12: **Identifying switching domains. (a)** The EBIC image (PUNDp7 − PUNDp0)/2, *i.e.,* the difference (Fig. 3a −Fig. 3c)/2. (The background subtraction performed in Fig. 3 is irrelevant, since it drops out in the difference.) The histogram indicates how many pixels in the image generated a given EBIC. **(b)** A binary mask generated by first thresholding the image (a) at the EBIC indicated by the vertical red line in the histogram, and then requiring that at least 5 above-threshold pixels be connected. This mask (b) shows what regions are switched from $P_\downarrow$ to $P_\uparrow$ by the 7 V var voltage pulses during PUNDp7. **(c)** Using the PUNDp data (Movie M2), a stack of 16 masks exactly like (b) is made, except that neighboring var voltage values are subtracted instead of the first and the last. This stack of binary masks is then summed and thresholded such that values not equal 1 are set to zero. The mask (c) is the result of an AND operation between the switch mask (b) and this sum mask. It thus shows the pixels that evidence one and only one E_{c+} in the PUNDp dataset. **(d,e,f)** The equivalent image/masks for the NDPUn data. The mask (f) shows the pixels that evidence one and only one E_{c-} in the NDPUn dataset. See Fig. S13.

The dark region in the lower right corner of both (a) and (b) is the edge of the capacitor. It is not ferroelectric, but it temporarily acquires some electric field during imaging due to charging by the beam.

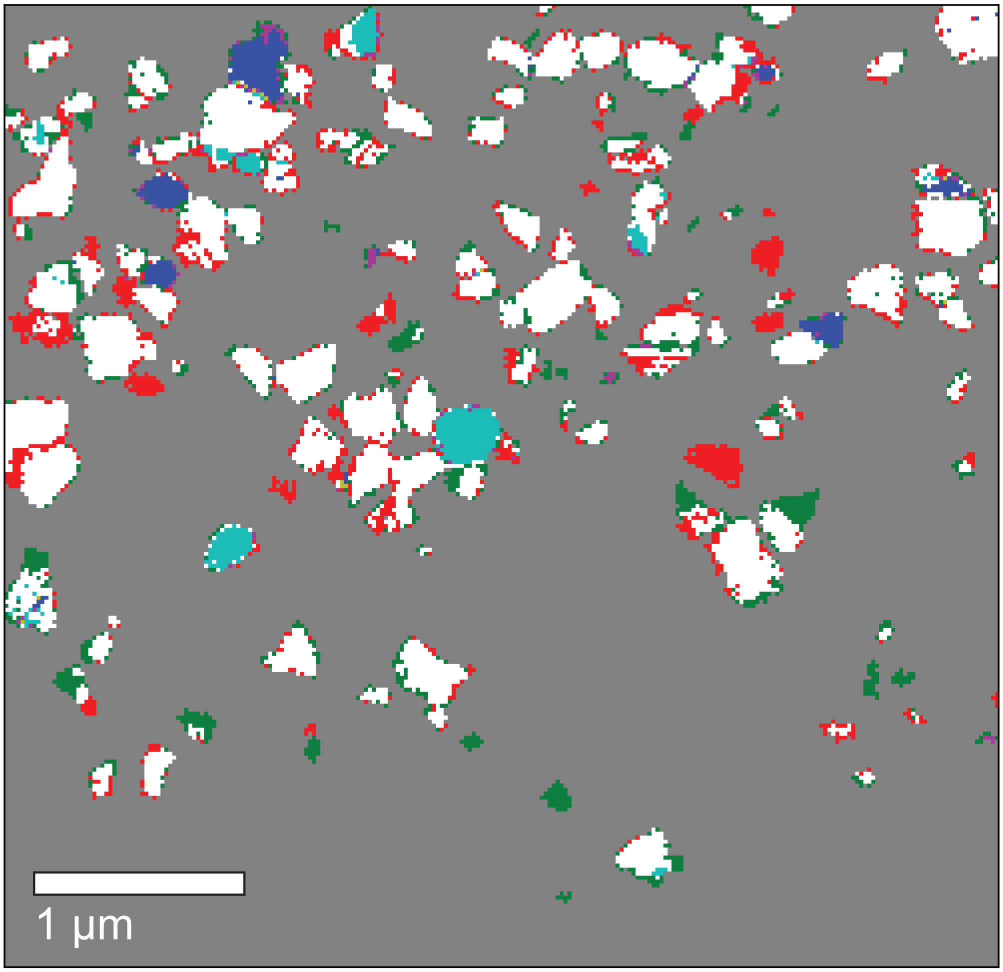

colors	PUNDp	NDPUn	pixels	%
	1	1	7025	11.7
	1	0	1821	3.0
	0	1	1558	2.6
	0	2	500	0.8
	2	0	510	0.8
	1	2	426	0.7
	2	1	464	0.8
	2	2	13	0.0

Figure S13: **Domain-scale switch count.** This map indicates the switch count of each area in the Fig. 3 dataset (Movie M2) by color. While most switchable domains (57%, white) show one E_{c+} and one E_{c-}, a non-negligible percentage demonstrate less consistent behavior. For instance, 3%, or 426 pixels (blue) of the 12,317 switching pixels, show one E_{c+} and two E_{c-}'s. Of the pixels that switch, 36% failed to show one of the $E_{c\pm}$; and 7% show both, but more than one of at least one of them. Only the pixels colored white here (representing an AND operation between Figs. S12c and S12f) are used to generate Fig. 3d.

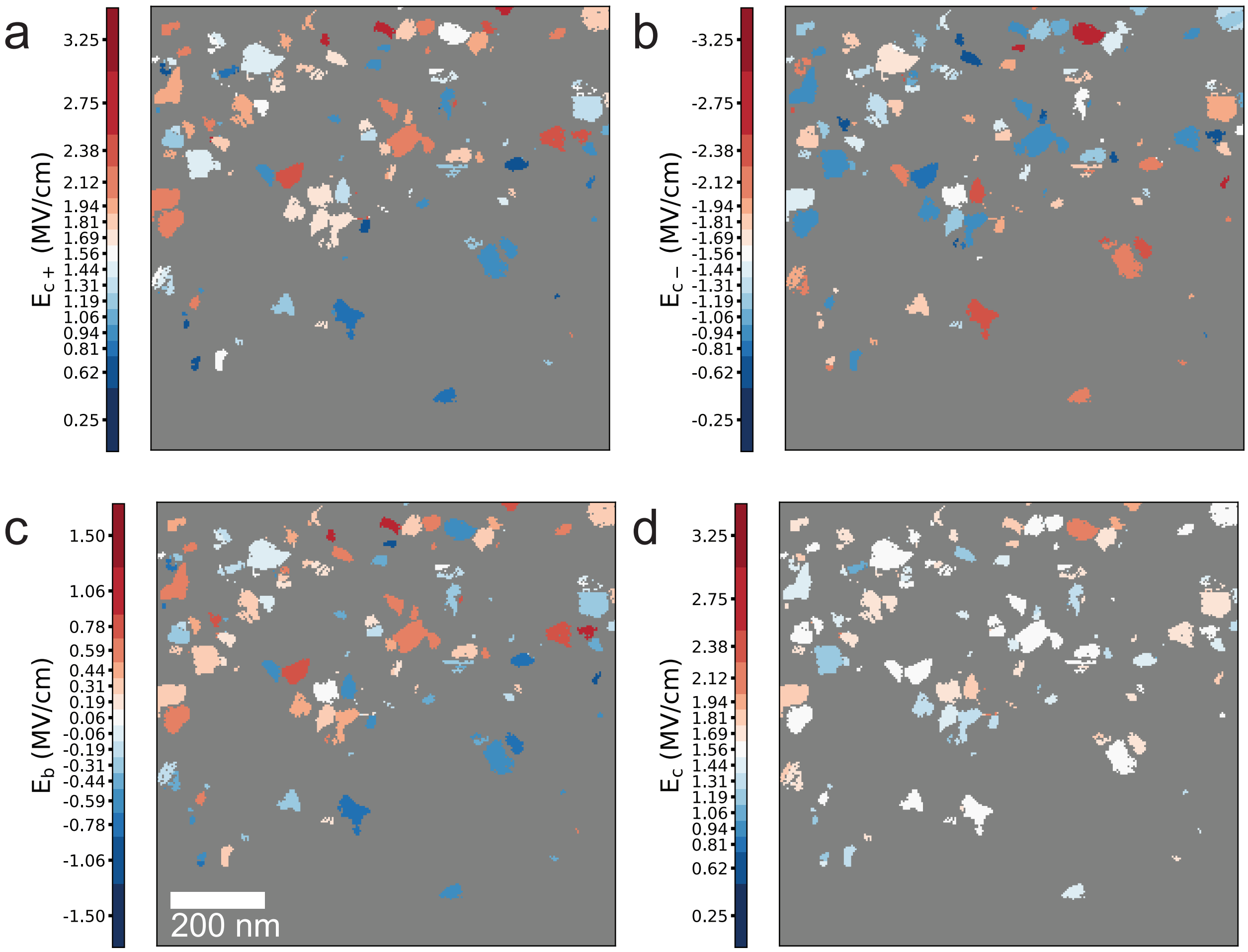

Figure S14: **Domain-scale $E_{c\pm}$, E_b, and E_c maps.** The coercive fields needed to switch the domains **(a)** up and **(b)** down for the dataset of Figs. 3–4 and Movie M2. The E_c maps are filtered such that (a) and (b) show the same domains; only pixels demonstrating one and only one coercive field for each polarity are shown. These maps show the same data as Fig. 3d and Fig. 4b, but sliced in a different direction. While the negative correlation between the magnitudes of E_{c+} and E_{c-} is less obvious here, it is more obvious that the switching unit is the domain, not the pixel. In other words, many neighboring pixels switch simultaneously. The $E_{c\pm}$ maps (a) and (b) are combined in the linear combinations **(c)** $E_b \equiv (E_{c+} + E_{c-})/2$ and **(d)** $E_c \equiv (E_{c+} - E_{c-})/2$. The even linear combination (c) shows that the effective positive/negative bias is strongly bimodal; the E_b are mostly positive or negative, with few near zero. The odd linear combination (d) shows again that, once the bias is subtracted out, the resulting E_c distribution is narrow and centered around 1.6 MV/cm. (The color scale for all four maps a–d has the same full range of 3 MV/cm, but the offsets are different.) No obvious correlations with domain size are evident. Based on our experience and what we can see in the bright field STEM images outside the capacitor, we expect that the HZO grain size is comparable to the 20-nm HZO film thickness. (The 50 nm of TaN electrode interferes with mapping the grains inside the capacitor.) Correlating the HZO grains with the ferroelectric domains will be the subject of future work.

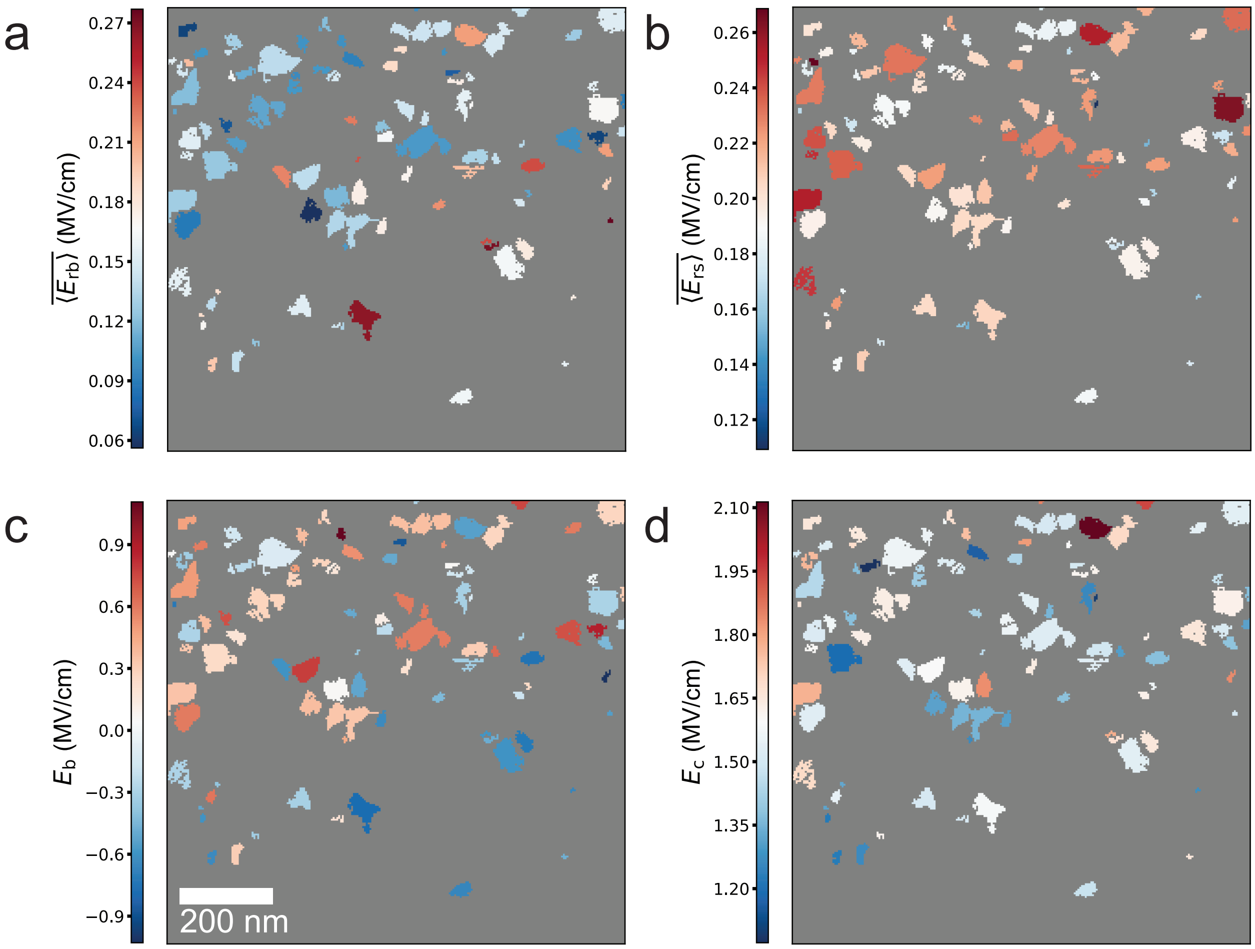

Figure S15: **Domain-scale $\overline{\langle E_{\mathrm{rb}} \rangle}$, $\overline{\langle E_{\mathrm{rs}} \rangle}$, E_b, and E_c maps.** The domain-scale STEM EBIC dataset (Movie M2) is analyzed (Movie M4) to quantify **(a)** $\overline{\langle E_{\mathrm{rb}} \rangle}$ and **(b)** $\overline{\langle E_{\mathrm{rs}} \rangle}$. The same analysis also returns **(c)** E_b and **(d)** E_c, which were previously determined in a slightly different way (Fig. S14c–d). The two methods agree. This map-style display reveals spatial correlations, but none are very obvious. However, comparing the each domain's different fields on a domain-by-domain basis (Movie M4, Fig. S18) shows that $\overline{\langle E_{\mathrm{rb}} \rangle}$ and E_b are negatively correlated. See Fig. S20 and Movie M5 for the results of a similar analysis, but without masking or intra-domain spatial averaging.

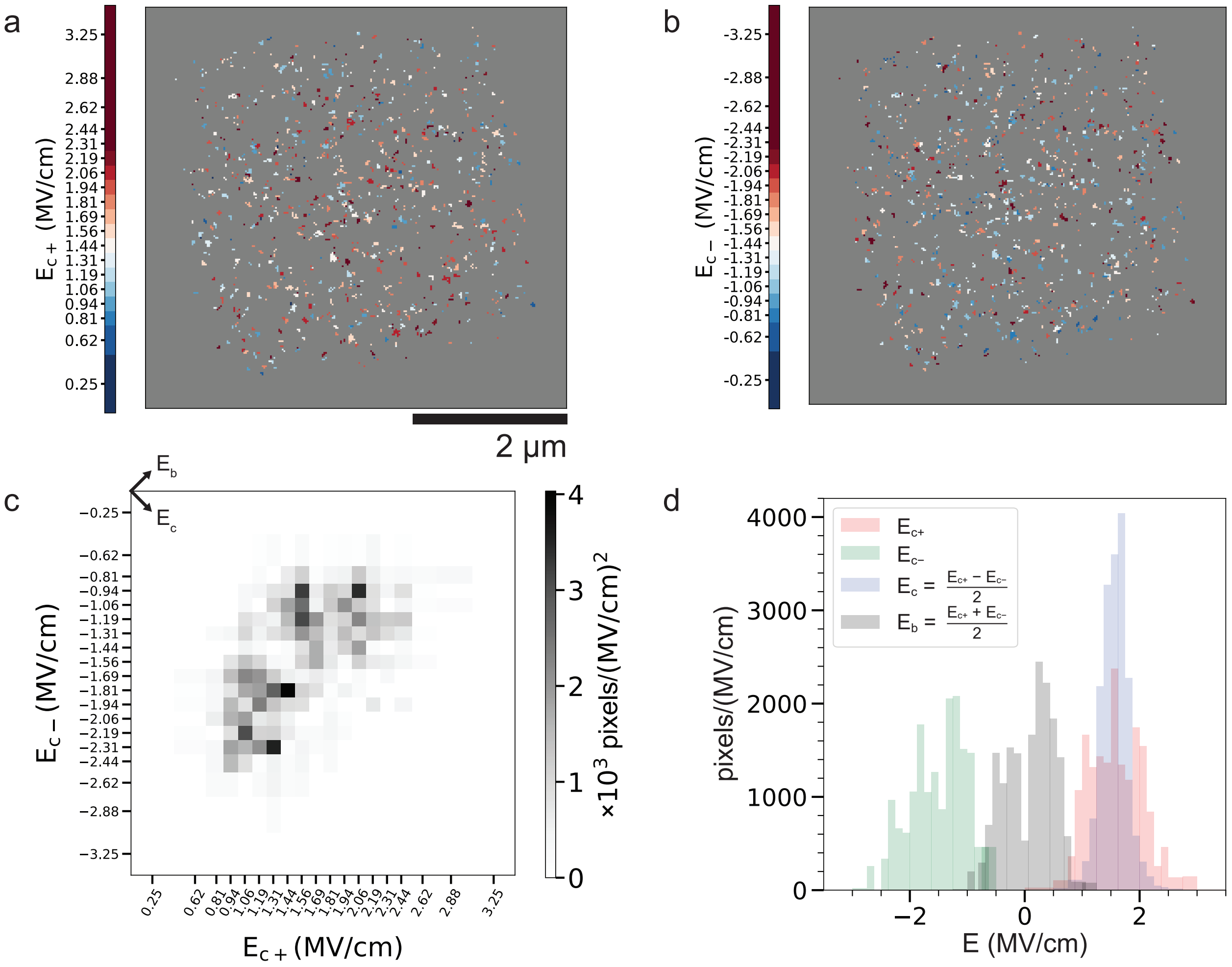

Figure S16: **Device scale $E_{c\pm}$ maps and distribution.** The coercive fields needed to switch the domains **(a)** up and **(b)** down for the dataset of Fig. 2 and Movie M1. The $E_{c\pm}$ maps are filtered such that (a) and (b) show the same domains; only pixels demonstrating one and only one coercive field for each polarity are shown. The $E_{c\pm}$ maps generate a 2D distribution **(c)** similar to Fig. 3d showing an obvious negative correlation between the magnitudes of E_{c+} and E_{c-}. A histogram **(d)** shows the number of pixels (or area) on the capacitor that can be switched at different bias fields. At this magnification frame-to-frame spatial alignment is less precise, but the distribution of the $(E_{c+} - E_{c-})/2$ linear combination is still narrower than the others. We find $E_{c+} = 1.6 \pm 0.5$, $E_{c-} = -1.5 \pm 0.5$, $E_c = 1.6 \pm 0.2$, and $E_b = 0.1 \pm 0.4$, all in MV/cm. Thus, despite the 6× difference in magnification, the device-scale dataset finds the same coercive and bias field values as the domain-scale dataset. It thus also supports the conclusion that local variations are affecting the positive/negative coercive field bias E_b and not the polarity-independent coercive field E_c.

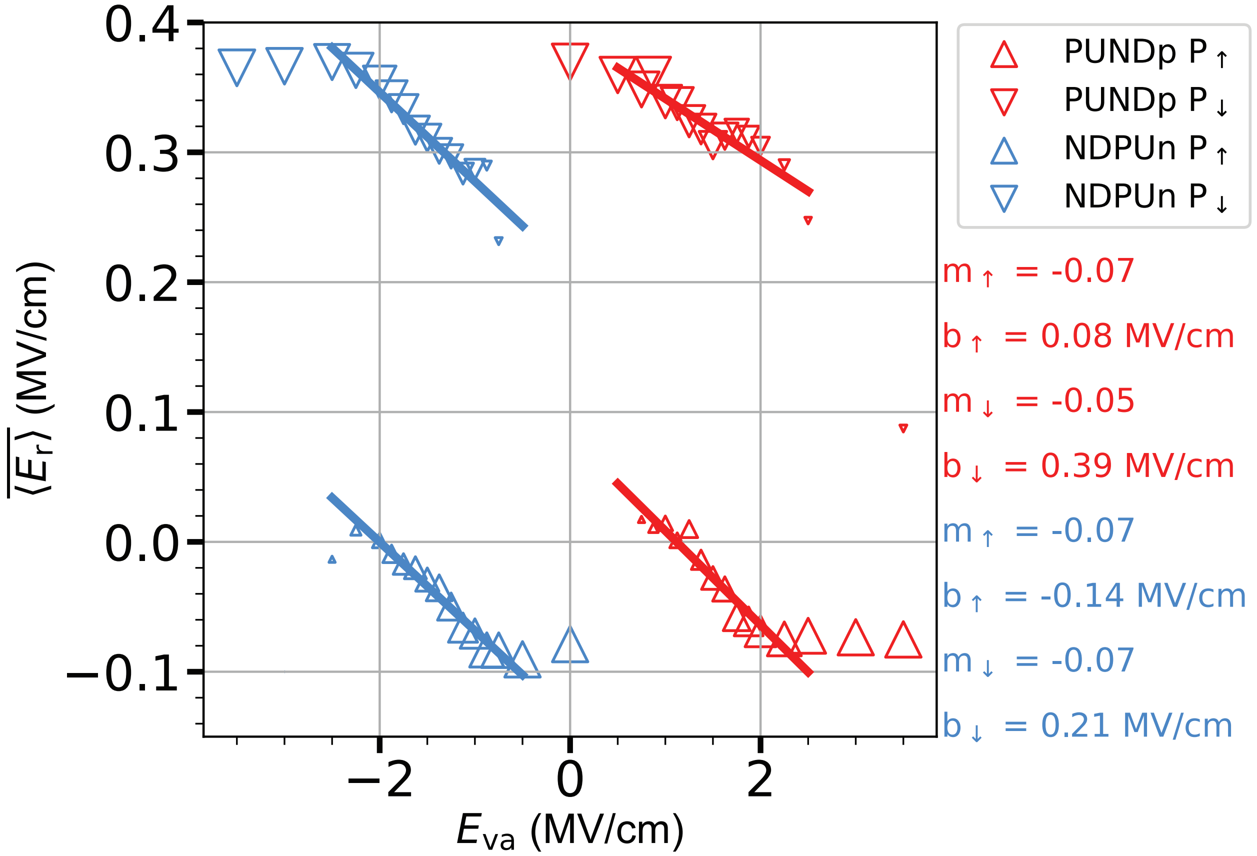

Figure S17: **Shift in $\langle E_r \rangle$ for $P_\uparrow$ and $P_\downarrow$ separately.** Movie M3 analyzes the switching regions for each E_{va} in the Fig. 3 dataset, fitting the $P_\uparrow$ and $P_\downarrow$ distributions to Gaussian functions. As the movie makes clear, decreasing $|E_{va}|$ in the PUNDp (NDPUn) sequence moves successively more and more population from the $P_\uparrow$ ($P_\downarrow$) state to the $P_\downarrow$ ($P_\uparrow$) state. It is also clear that, as the populations in each state change, the mean $\overline{\langle E_r \rangle}$ also shifts. Here the mean $\overline{\langle E_r \rangle}$, averaged over the domains that switch once (Fig. S13) from each $P_\updownarrow$ state separately, is plotted as a function of E_{va}. Populations too small to display have been dropped. The plot marker areas are proportional to the normalized populations Φ determined by the fit. These data show, for instance, that the domains with the largest remanent background fields $\langle E_{rb} \rangle$ (note that the $\overline{\langle E_r \rangle}$-axis is inverted) switch from $P_\uparrow$ to $P_\downarrow$ at the smallest E_{va}. These data, here shown as a function of E_{va}, are the same as those plotted in Fig. S18 domain-by-domain.

If $\langle E_{rb} \rangle$ were constant or uncorrelated with the coercive field bias E_b, these means would not shift. The ferroelectric switching current $I(V)$ is a measurement of the domain switching distribution as a function of the applied field E_a. This plot demonstrates that this distribution (the red curves in Fig. 1 and Fig. 4a) is equivalently produced by partitioning according to the local background fields $\langle E_{rb} \rangle$. Thus, the measurable variations in the background fields are governing the width of the coercive field $E_{c\pm}$ distributions.

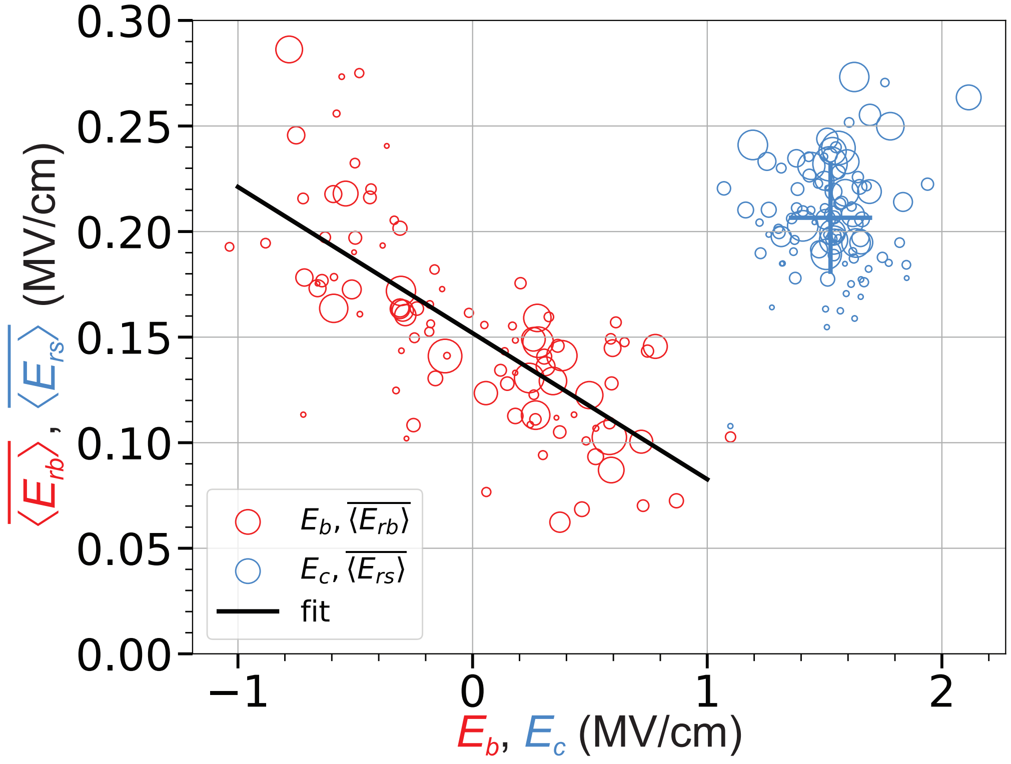

Figure S18: **Local background fields shift $E_{c\pm}$, domain scale.** The STEM EBIC dataset (Movie M2) can be analyzed to quantify the relationships between externally-applied E-fields and internal remanent E-fields. Each data point here represents a domain, with the area of the point proportional to the number of pixels in the domain. The domains plotted range in area from 5 pixels [$(8.5\text{ nm})^2$] to 277 pixels [$(63\text{ nm})^2$]. In the analysis below the domains are weighted equally, independent of their area. The x-axis E-fields are generated by the PUND function generator, and the particular values are found by determining at what fields $E_{c\pm}$ the domain switches with STEM EBIC (Movie M4). These values have two components: E_c, which describes the unbiased coercive field, and E_b, which describes the coercive field bias ($E_{c\pm} = E_b \pm E_c$). The y-axis electric fields are remanent fields $\overline{\langle E_r \rangle}$ in the capacitor that are measured with STEM EBIC. The fields $\overline{\langle E_r \rangle} = \overline{\langle E_{rb} \rangle} \pm \overline{\langle E_{rs} \rangle}$ also have two components: $\overline{\langle E_{rs} \rangle}$ is the portion that is switchable and $\overline{\langle E_{rb} \rangle}$ is the (non-switching) background field.

We plot $[E_c, \overline{\langle E_{rs} \rangle}]$ (blue) and $[E_b, \overline{\langle E_{rb} \rangle}]$ (red). The coercive field E_c and the switching field $\overline{\langle E_{rs} \rangle}$ do not show any obvious correlation. We find $E_c = 1.5 \pm 0.2$ MV/cm, in good agreement with the analysis of Fig. 4, which was done in a slightly different way. For the switching field we find $\overline{\langle E_{rs} \rangle} = 0.21 \pm 0.03$ MV/cm, in good agreement with the analysis of Fig. 2, which again was done in a slightly different way. The blue cross indicates these means and standard deviations graphically.

More interesting is the evident anti-correlation between E_b and $\overline{\langle E_{rb} \rangle}$. The bias field E_b describes how much the $P(E)$ loop is shifted along the E-axis, so a positive E_b means that *more* positive applied fields E_a are required to generate a switch from $P_\downarrow$ to $P_\uparrow$. We see that domains with more positive E_b have a more negative remanent background field $\overline{\langle E_{rb} \rangle}$ (fit: slope −0.07, offset 0.15 MV/cm). We expect such a relationship: to induce a positive switch, E_a must be larger in the presence of a more negative background field (Fig. S10). We also expect the slope magnitude to be less than unity, as STEM EBIC measures the screened background fields and the PUND function generator is removing that screening to affect a switch. These results demonstrate that not only can STEM EBIC identify the coercive fields for individual domains in polycrystalline ALD HZO, it can also quantify the background fields that produce the dispersion in those same individual domains.

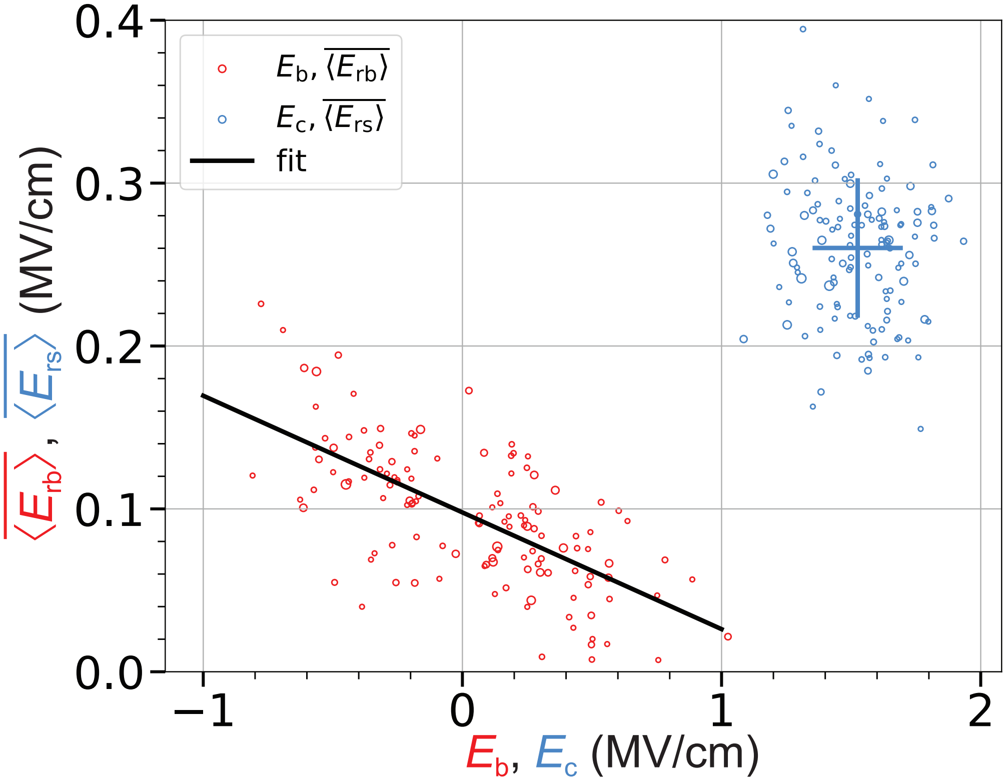

Figure S19: **Local background fields shift $E_{c\pm}$, device scale.** This figure shows the same analysis as that of Fig. S18, but here applied to the device-scale dataset (Movie M1) instead of the domain-scale dataset (Movie M2). As indicated by the size of the data points, the individual domains appear as just a few pixels in the device-scale images. The domains plotted range in area from 5 pixels [(50 nm)^2] to 19 pixels [(100 nm)^2] (again domains of fewer than 5 pixels are not considered), with the 5 pixel domains being the most common.

Nonetheless, the measurements of the coercive fields E_c, the bias fields E_b, and the switching fields $\overline{\langle E_{\rm rs}\rangle}$ are consistent with those found at the higher magnification. In this device-scale data we find $E_c = 1.5 \pm 0.2$ MV/cm and $\overline{\langle E_{\rm rs}\rangle} = 0.27 \pm 0.04$ MV/cm, in agreement with the results for the domain-scale data (Figs. 4 and S18). The anti-correlation between E_b and $\overline{\langle E_{\rm rb}\rangle}$ found here (fit: slope -0.07), is also in agreement with that found in the other dataset.

The fit offset 0.10 MV/cm is significantly lower than that found in Fig. S18. This discrepancy is not unexpected, and it points to opportunities for optimization. It indicates, just as the device-scale $(P_\uparrow + P_\downarrow)/2$ STEM EBIC images do (*e.g.,* Fig. 2d and Fig. S8d), that the remanent background fields $\overline{\langle E_{\rm rb}\rangle}$ are varying across the capacitor.[3] These concordant results provide another example of how the magnification-independence of the STEM EBIC contrast mechanisms makes it possible to conduct rapid device-scale surveys that return trustworthy and useful measurements of domain-scale properties.

[3] Because they do not switch, the background remanent fields $\langle E_{\rm rb}\rangle$ are intrinsically more difficult to determine than the switching remanent fields $\langle E_{\rm rs}\rangle$. An E-field-independent contribution to $(I_{\rm top} - I_{\rm bot})/2$, such as a SEEBIC effect, cannot be distinguished from a true background E-field. We have such a contribution outside the capacitor (Fig. S8), and we suspect a slightly larger one inside the capacitor. In fact, it might be most appropriate to subtract 0.1 MV/cm and 0.15 MV/cm from the $\langle E_{\rm rb}\rangle$ values here and in Fig. S18, respectively. Regardless, it is the spatial variation of $\langle E_{\rm rb}\rangle$ from its mean, not the mean itself, which is the most interesting for its effect on E_b.

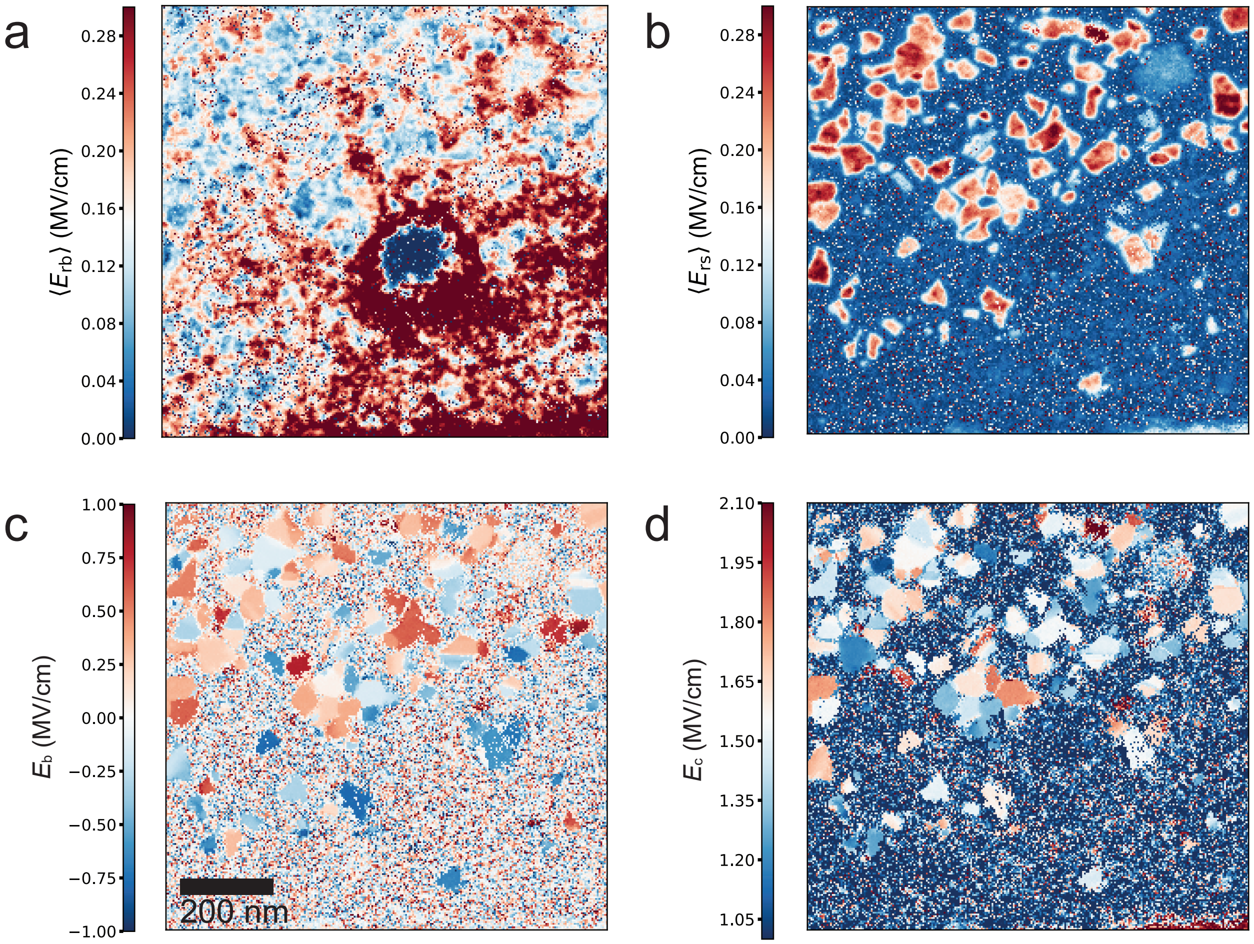

Figure S20: **Domain-scale** $\langle E_{\mathrm{rb}} \rangle$, $\langle E_{\mathrm{rs}} \rangle$, E_b, **and** E_c **maps, pixel-by-pixel.** This figure is identical to Fig. S15, except that there is no masking and no intra-domain spatial averaging. The STEM EBIC signal-to-noise ratio is good enough to support the tanh analysis of Movie M4 at the single-pixel level wherever there is a switching field. Of course, where there is no switching field, the fitting returns the mean signal for **(a)** $\langle E_{\mathrm{rb}} \rangle$, zero for **(b)** $\langle E_{\mathrm{rs}} \rangle$, and only noise for **(c)** E_b and **(d)** E_c. For this figure $\langle E_{\mathrm{rs}} \rangle$ is defined as the field that is switching at or below $|E_{\mathrm{va}}| = 3.5$ MV/cm. Because of this definition, some regions pinned at 3.5 MV/cm (but presumably switchable at larger fields) appear with an artificially large $\langle E_{\mathrm{rb}} \rangle$.

This field of view shows structure that has not been analyzed or even mentioned yet in this paper. For instance, a variety of domain walls are evident that the one-and-only-one-switch mask (Fig. S13) applied to Fig. S15 hides. The analysis presented in this work has mined only a fraction of this multidimensional dataset.
